# Investigating the Causal Relationship and Shared Genetic Basis Between Major Depression Disorder and Eight Types of Gastrointestinal Diseases

**DOI:** 10.1155/genr/6931243

**Published:** 2025-12-14

**Authors:** Fu Sun, Haochang Li, Bin Gong, Shirong Hui, Ran He, Meijie Yu, Yihao Li, Sheng Yang, Peng Huang

**Affiliations:** ^1^ The People’s Hospital of Danyang, Affiliated Danyang Hospital of Nantong University, Zhenjiang, 212300, China; ^2^ Department of Epidemiology, Center for Global Health, School of Public Health, National Vaccine Innovation Platform, Nanjing Medical University, Nanjing, 211166, China, njmu.edu.cn; ^3^ Department of Biostatistics, Center for Global Health, School of Public Health, National Vaccine Innovation Platform, Nanjing Medical University, Nanjing, 211166, China, njmu.edu.cn

**Keywords:** causal relationship, gastrointestinal diseases, major depression disorder, shared genetic basis

## Abstract

**Background:**

Previous studies on the causal relationship or shared genetic basis between major depression disorder (MDD) and gastrointestinal (GI) diseases covered either a limited range and not comprehensive enough.

**Methods:**

We used linkage disequilibrium score regression (LDSC) to estimate the heritability for nine traits and the genetic correlation (*r*_*g*_) between MDD and eight GI diseases, respectively. We further conducted a two‐sample Mendelian randomization (MR) analysis to identify the causal relationship between MDD and eight GI diseases. Finally, based on the result of MR, we performed stratified LDSC (S‐LDSC) to estimate the partitioned heritability and significantly enriched tissues, transcriptome‐wide association study (TWAS) to define shared genes, and colocalization analysis to define the pleiotropic single‐nucleotide polymorphisms (SNPs) and genes.

**Results:**

For the heritability, heritability of all nine traits was significant. For genetic correlation, six GI diseases showed significant correlations with MDD. For the result of MR, we revealed the causal relationship between MDD and acute appendicitis (OR = 1.09), irritable bowel syndrome (IBS) (OR = 1.14), and ulcer of esophagus (OR = 1.24). Additionally, we found no significant overlapping tissues after S‐LDSC. Finally, we defined three shared genes: *PRSS16, ZNF602P,* and *ZNF204P* by TWAS and nine pleiotropic genes *C4A*, *FLOT1*, *LINC00243*, *MICB*, and *PRSS16* by colocalization analysis between MDD and acute appendicitis.

**Conclusions:**

Our findings provided the evidence of genetic association, causal relationship, and shared pleiotropic genes between MDD and GI diseases especially acute appendicitis, offering new insights into our understanding of their shared genetic basis.

## 1. Introduction

Gastrointestinal (GI) diseases are a collection of diseases originating from digestive dysfunctions, posing a significant threat to human health [1]. The pathogenesis of GI diseases is exceedingly complex, involving multiple contributing factors. Based on cohort and case‐control studies, emerging studies have suggested that major depression disorder (MDD) is a significant risk factor for certain GI disorders [2–4], including irritable bowel syndrome (IBS) [5], gastroesophageal reflux disease (GORD) [6], and peptic ulcers [7]. However, observational studies face limitations, such as confounding factors and the lack of randomized design and genetic basis evidence [8]. Therefore, stronger evidence especially the genetic evidence needed to confirm the causal relationship between MDD and GI diseases.

In the past 20 years, genome‐wide association studies (GWASs) have effectively identified the association between genetic variants and numerous complex traits [9], providing novel insights into the pathogenesis of diseases [10]. In terms of MDD, the two largest GWASs discovered 44 and 102 risk loci associated with the disorder, respectively [11, 12]. Amount summary statistics of GWAS provides the researcher more opportunity to investigate the heritability, stratified heritability, causal inference between two complex diseases, and the association between gene expression trait based on single‐nucleotide polymorphisms (SNPs). Linkage disequilibrium score regression (LDSC) estimates trait heritability and global genetic correlations between traits [13]. The stratified LDSC (S‐LDSC) explores heritability enrichment in specific cell and tissue types from SNPs [14]. Mendelian randomization (MR) uses genetic variants to explain causal relationships between exposures and outcomes [15]. Transcriptome‐wide association study (TWAS) integrates GWASs with expression quantitative trait loci (eQTLs) to investigate the relationship between tissue‐specific gene expression and trait variation [16]. Colocalization analysis employs Bayesian statistical methods to detect shared genetic variants in common genomic regions across traits [17].

Several studies have used these methods to explore the shared genetic basis of MDD and GI diseases. For example, a two‐sample MR study has demonstrated a causal relationship between MDD and an increased risk of 12 out of 24 GI diseases examined [18]. Another comprehensive analysis has further revealed the pleiotropic genes and the role of the gut–brain axis in mediating the relationship between MDD and GORD, IBS, peptic ulcer disease (PUD), and nonalcoholic fatty liver disease (NAFLD) [19]. However, while the former study covered a wide range of diseases, it only explored the causal relationship; the latter study employed more comprehensive methods but was limited to a few GI diseases. Therefore, it is necessary to investigate in depth the complex genetic mechanisms underlying the comorbidity between MDD and GI diseases, to provide additional insights into the prevention of GI diseases.

**Figure 1:**
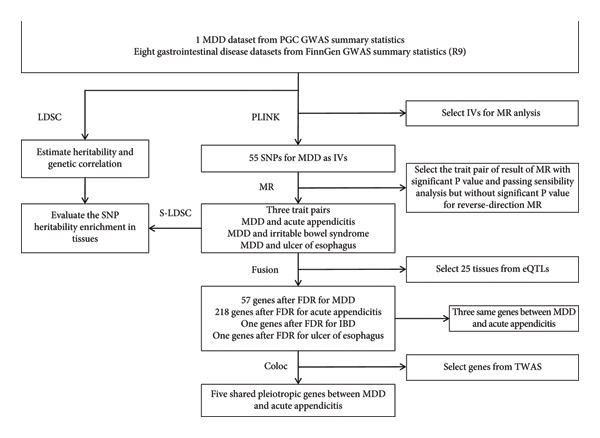
The analytical flowchart in our study. We collected MDD summary datasets from PGC and GI diseases from FinnGen R9. First, we used LDSC to estimate heritability and genetic correlation. Then, we used MR and a series of sensitivity analyses to define the causal relationship between MDD and each GI disease. Finally, integrating gene expression and eQTL summary statistics of GTEx, we used S‐LDSC, FUSION, and coloc to define the enrichment tissues, shared significant genes, and shared pleiotropic genes, respectively. MDD, major depression disorder; GI, gastrointestinal; LDSC, linkage disequilibrium score regression; MR, Mendelian randomization; eQTL, expression quantitative trait loci; S‐LDSC, stratified linkage disequilibrium score regression.

In our study, we adopted a comprehensive approach integrating various methodologies including LDSC, MR, S‐LDSC, TWAS, and colocalization analysis to elucidate the shared genetic basis and potential causal relationship underlying MDD and eight GI diseases (i.e., Crohn disease, gastric ulcer, GORD, gastroduodenal ulcer, ulcerative colitis, acute appendicitis, IBS, and ulcer of esophagus). We aimed to deeply understand of the shared genetics between MDD and GI diseases. The detailed flowchart in this study is shown in Figure 1. This study was conducted using publicly available GWAS summary statistics; thus, individual‐level data were not accessible, and a conventional participant flow diagram illustrating individual inclusion and exclusion is not applicable. The comprehensive analytical workflow, spanning from data acquisition to final result generation, is visually summarized in Figure 1.

## 2. Methods

### 2.1. Data Collection and Processing

In the analysis, we used three kinds of data resources: summary statistics for the MDD, summary statistics for eight GI diseases, and reference LD panel. Specifically, the GWAS summary statistics for MDD were obtained from a meta‐analysis conducted by the Psychiatric Genomics Consortium (https://pgc.unc.edu/), which included case–control studies from multiple cohorts of European ancestry [11]. The sample comprised 135,458 cases and 344,901 controls. The PGC MDD GWAS meta‐analysis reported minimal evidence of heterogeneity across the contributing cohorts, as detailed in the original publication. The summary statistics of eight GI diseases were downloaded from FinnGen GWAS summary statistics (R9) (https://storage.googleapis.com/finngen-public-data-r9/summary_stats/), a large Finnish cohort study that combines genetic data with digital health record data from national health registries of European ancestry [20], including Crohn disease, gastric ulcer, GORD, gastroduodenal ulcer, ulcerative colitis, acute appendicitis, IBS and ulcer of esophagus. The specific number of cases and controls for each GI disease is provided in Table 1. The underlying study design for both data sources is case–control. Power calculations for the MR analysis were not performed a priori as the analysis utilized publicly available summary statistics with fixed sample sizes. However, the large sample sizes for most traits (e.g., MDD: *N* = 480,359; acute appendicitis: *N* = 375,028) provide substantial power to detect genetic correlations and causal effects of moderate magnitude. Missing genetic data in the GWAS summary statistics were handled by the original studies (PGC and FinnGen). In our analysis, we carried out strict quality control for these data. Specifically, we filtered out SNPs (1) with minor allele fraction (MAF) < 0.01, (2) with INFO < 0.3, (3) that are not included in the reference panel, and (4) that are duplicated, which are standard quality control procedures to minimize the impact of poorly imputed or rare variants​ [21–23]. Phenotypic data were complete in the summary statistics as they represent case–control status. In this study, we considered 1000 Genomes Project Phase3 European population SNPs as the reference panel. For the reference panel, we used 503 EUR individuals to estimate the LD matrix [24].

**Table 1:** Summary for the MDD and eight gastrointestinal diseases.

Disease	No. of cases	No. of controls	nSNPs	*h* ^2^	*p*
Major depressive disorder	135,458	344,901	8,710,666	0.0217	0.0014
Crohn disease	1655	375,445	9,199,253	0.0083	0.0016
Gastric ulcer	5935	320,387	9,198,650	0.0043	0.0014
Gastroesophageal reflux disease	26,184	320,387	9,198,761	0.0230	0.0018
Gastroduodenal ulcer	9216	320,387	9,198,769	0.0062	0.0015
Ulcerative colitis	5034	371,530	9,199,218	0.0167	0.0026
Acute appendicitis	28,745	346,283	9,199,249	0.0121	0.0019
Irritable bowel syndrome	9323	301,931	9,198,970	0.0092	0.0017
Ulcer of esophagus	2064	320,387	9,198,631	0.0050	0.0014

### 2.2. Heritability and Genetic Correlation

We used LDSC to estimate the global heritability for each disease and the genetic correlation (*r*_*g*_) between MDD and eight GI diseases, respectively. Also, we used S‐LDSC to estimate the partitioned heritability and significantly enriched tissues. Specifically, integrated the summary statistics of eQTLs from GTEx V8 (http://gusevlab.org/projects/fusion/#gtex-v8-multi-tissue-expression) [25], S‐LDSC defined the associated tissues among 25 selected tissues using the top significant genes [14]. Considering the MDD and GI diseases, we selected 15 tissues from the nervous system (i.e., brain amygdala, brain anterior cingulate cortex, brain caudate basal ganglia, brain cerebellar hemisphere, brain cerebellum, brain cortex, brain frontal cortex, brain hippocampus, brain hypothalamus, brain nucleus accumbens basal ganglia, brain putamen basal ganglia, brain spinal cord cervical, brain substantia nigra, and nerve tibial, pituitary), nine tissues from digestive system (i.e., colon sigmoid, colon transverse, esophagus gastroesophageal junction, esophagus mucosa, esophagus muscularis, liver, pancreas, small intestine terminal ileum, and stomach), and whole blood. We defined the differentially expressed genes (DEGs) by the Wilcoxon test and set the SNP annotation as 1 if it located within 100 KB of DEGs. Following Ref. [26], we added the focal functional categories to the baseline model and model of top 5% or 10% genes. Finally, we used S‐LDSC to estimate the enrichment for each tissue.

### 2.3. MR Analysis

To define the causal relationship between MDD to eight GI diseases, we used the two‐sample MR analysis. Given that the exposure and outcome data were derived from distinct consortia and predominantly nonoverlapping populations, the overlap of individuals between these two samples is expected to be minimal or null, thus satisfying the key assumption of two‐sample MR. The crucial step of MR was performed to select genetic variants as instruction variables (IVs) for exposure. Following Refs. [27–29], we retained SNPs with (1) strong association (*p* value < 1 × 10^−5^) in MDD with *r* ^2^ less than 0.001 in the range of 10 Mb and (2) strong IVs with F‐statistics > 10 [30]. To ensure that the genetic variant–exposure associations were comparable between the two samples, we performed rigorous data harmonization by aligning all effect alleles to the same reference strand, filtering out SNPs with palindrome alleles and SNPs that were strongly correlated with outcome (*p* < 1 × 10^−5^), thereby minimizing biases arising from differences between consortia. To obtain robust result, we carried out five models: MR‐Egger method [31], weighted median (WM) method [32], inverse variance weighting (IVW) method [33], simple mode method, and weighted mode method [34]. We mainly considered the IVW method as the main result [33, 35].

In parallel, we performed three sensitivity analyses: (i) heterogeneity test—to test the heterogeneity among IVs, (ii) MR pleiotropy residual sum and outlier (MR‐PRESSO)—to find outliers and test the level of pleiotropy, and (iii) leave‐one‐out (LOO) test—to observe whether the result significantly changed after removing each SNP [36, 37]. For these sensitivity analyses, we considered 0.05 as the significant level of statistic tests. In addition, we performed reverse‐direction MR to assess potential reverse causal effects of GI diseases on MDD. Parameters of selecting IVs and analysis procedure, as well as sensitivity analyses are the same as those in the forward direction MR analysis.

### 2.4. TWAS

Based on the results of MR, we only defined shared significant genes among the causal traits. FUSION, defining the top eQTL with the best prediction model, was used to fit TWAS [38]. We used the same eQTL summary statistics from 25 tissues and the same reference panel in S‐LDSC. For each trait pair, we needed the shared genes with statistical significance.

### 2.5. Colocalization

Integrating summary statistics of eQTLs, we used the colocalization analysis to define the pleiotropic SNPs for each trait. The colocalization analysis includes five model assumptions: (1) H0: no association, (2) H1: only association with Trait 1, (3) H2: only association with Trait 2, (4) H3: association with Trait 1 and Trait 2, distinct causal variants, and (5) H4: association with Trait 1 and Trait 2, shared causal variants [39]. Following Ref. [40], we considered the result of PP.H4 above 0.75 as significant results. The eQTLs we used in the colocalization analysis are consistent with those used in TWAS. We selected around 1 MB of the starting location of the significant candidate genes found in TWAS as the candidate region for the colocalization analysis.

### 2.6. Statistical Analysis

We used LDSC (Version 1.0.1) to estimate heritability (*h* ^2^), genetic correlation, and tissue type of SNP heritability enrichment in GWAS summary statistics. We used *TwoSampleMR* (Version 0.5.7) to perform two‐sample MR analysis and *coloc* (Version 5.2.3) to perform the colocalization analysis. We used *FUSION* (Version 0.0.1) to perform TWAS. All the analyses are performed by R software (Version 4.3.0). We performed multiple corrections to the results of genetic correlation, MR (including direct and reverse) and TWAS by using the false discovery rate (FDR) [41]. We considered 0.05 as the significant level of our statistic tests as well as FDR correction.

## 3. Results

### 3.1. Heritability and Genetic Correlation

**Figure 2:**
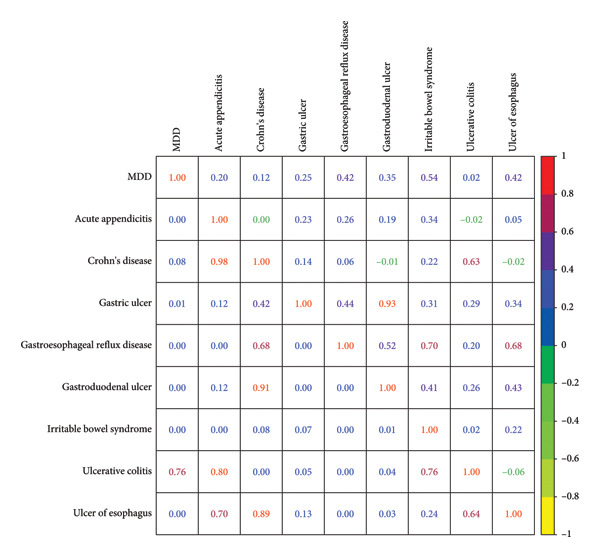
The genetic correlation of nine traits using linkage disequilibrium score regression. The heat map shows *r*_*g*_ (lower triangle) and its *p* value (upper triangle). The different colors represent different values of *r*_*g*_ and *p*. The red color shows a more positive correlation, while the yellow color shows a more negative correlation.

Summary statistics for the major depressive disorder and eight GI diseases, including sample sizes and heritability estimates, are presented in Table 1. We estimated the heritability for nine traits (Table 1) and genetic correlation (Figure 2 and Table S1) for eight trait pairs. For the heritability, heritability of all nine traits was significant. Specifically, *h* ^2^ for MDD was 0.0217 with *p* = 0.0014. *h* ^2^ for the eight GI diseases ranged from 0.0167 (*p* = 0.0026) for ulcerative colitis to 0.043 (*p* = 0.0014) for gastric ulcer. For genetic correlation, there were six significant results between MDD and eight GI diseases and the results were still significant after FDR. Specifically, the genetic correlation between MDD and eight GI diseases ranged from 0.2003 (*p* = 0.0005) to 0.5446 (*p* = 5.71 × 10^−11^).

### 3.2. MR Analysis

We strictly followed the procedures in our methods, and we eventually obtained 55 IVs of MDD, which includes effect alleles, beta coefficients, standard errors, and *p* values on the log‐odds scale (Table S2). The mean F‐statistic for these instruments was 28.7 (range: 15.3–45.2), substantially above the conventional threshold of 10, indicating a low risk of weak instrument bias. Based on the IVW model, we defined MDD was a risky factor for five GI diseases by FDR threshold, including acute appendicitis (OR = 1.08, 95% CI: 1.02–1.15, *p* = 0.006, FDR = 0.024), gastroduodenal ulcer (OR = 1.10, 95% CI: 1.01–1.20, *p* = 0.029, FDR = 0.041), GORD (OR = 1.09, 95% CI: 1.02–1.16, *p* = 0.011, FDR = 0.028), IBS (OR = 1.13, 95% CI: 1.04–1.23, *p* = 0.005, FDR = 0.024), and ulcer of esophagus (OR = 1.24, 95% CI: 1.03–1.50, *p* = 0.025, FDR = 0.041) (Table S3).

To assess the validity of the MR assumptions, particularly, the absence of horizontal pleiotropy, we conducted comprehensive sensitivity analyses. For three causal associations that passed multiple testing correction (MDD on acute appendicitis, IBS, and esophageal ulcer), Cochran’s Q statistic indicated no significant heterogeneity among the variant‐specific estimates (all *p* > 0.05), suggesting that a fixed‐effect IVW model was appropriate. The MR‐Egger intercept test revealed no evidence of directional pleiotropy (all *p* > 0.05). Furthermore, the MR‐PRESSO global test detected no significant outliers (all *p* > 0.05), and the results remained consistent after correcting for potential outliers. The results of all direct sensitivity analyses are shown in Table S4. Besides, the WM and MR‐Egger methods yielded effect estimates that were directionally consistent with the IVW method, providing additional confidence in the primary findings. The F‐statistics for all instruments were well above 10 (mean: 28.7), minimizing concerns about weak instrument bias. We used the pictures including the forest plot, scatter plot, and funnel plot to show the results of the MR analysis. We displayed the result of MDD on acute appendicitis (Figure 3); others are shown in our Supporting information (Supporting information 1).

Figure 3The results of the MR analysis for MDD on acute appendicitis. (a) Forest plot for the MR effect size of each IV, with each point representing the causal effect by IVW if only using this SNP. The red line at bottom represents the effect size of the IVW method. (b) The scatter plot of causal effects of MDD on acute appendicitis. The vertical and horizontal black lines show the 95% CI of the estimated effect of IVs on MDD and acute appendicitis. The *x* axis represents the SNP effect on MDD, and the *y* axis represents the SNP effect on acute appendicitis. (c) The funnel plot of the causal effect of MDD on acute appendicitis. Each point represents the individual causal effect of every IV. The red line represents the causal effect estimate of the IVW method. MR, Mendelian randomization; MDD, major depression disorder; IV, instrumental variable; IVW, inverse variance weighting; SNP, single‐nucleotide polymorphism; CI, confidence interval.

**Figure:**
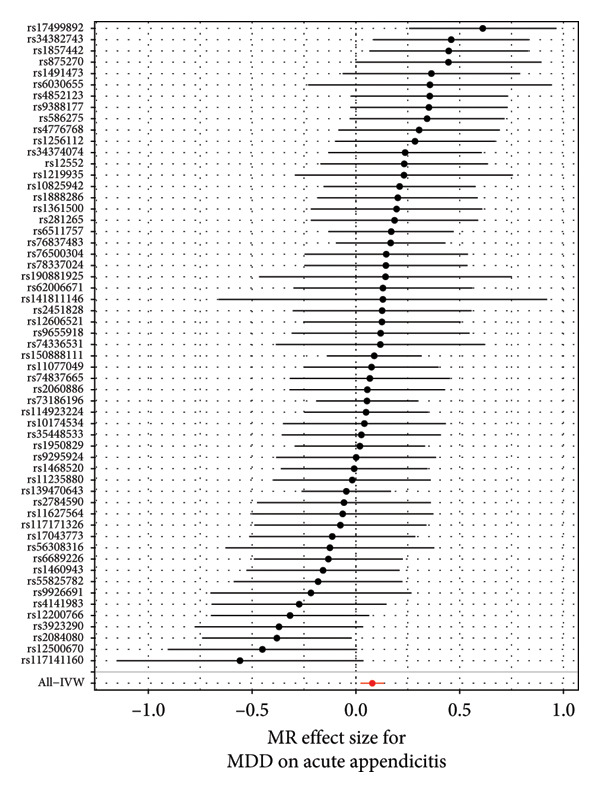
(a)

**Figure:**
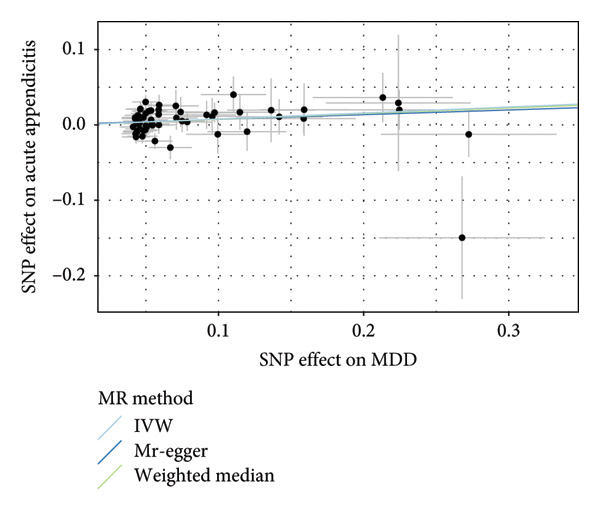
(b)

**Figure:**
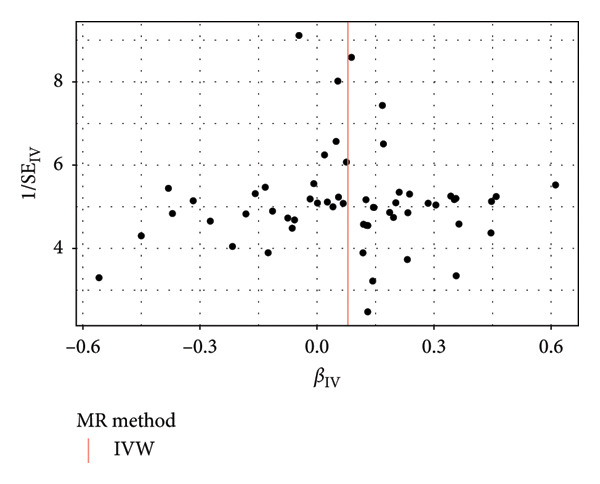
(c)

Finally, we performed reverse‐direction MR to explore whether there was potential reverse causality (Supporting information 2). Using the same criteria in MR, we obtained 47 IVs for acute appendicitis, 28 IVs for Crohn’s disease, 23 IVs for gastric ulcer, 50 IVs for GORD, 20 IVs for gastroduodenal ulcer, 22 IVs for IBS, 13 IVs for ulcer of esophagus, and 80 IVs for ulcerative colitis (Table S2). For the reverse result, we found GORD was a risky factor on MDD (OR: 1.09; 95% CI: 1.03–1.16; *p* = 0.005, FDR = 0.041), and other results did not show significance (Table S3). As for sensitivity analyses, GORD on MDD did not show obvious heterogeneity and pleiotropy, as well as outliers (*p* > 0.05), and all reverse sensitivity analyses are shown in Table S5.

### 3.3. SNP Heritability Enrichment of Different Tissues in GWAS Summary Statistics

**Figure 4:**
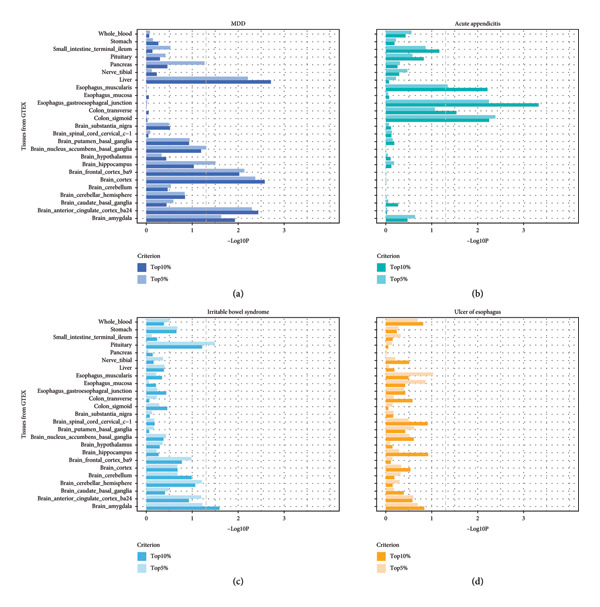
SNP heritability enrichment in different tissues. The *x* axis represents the negative log10 *p* values of coefficient *Z* scores, while the *y* axis represents the selected tissues from GTEx. The criterion in the legend represents the different defined focal functional categories, the dark color represents using top 10% of marker genes, while the light color represents 5% of selected marker genes. The gray dotted line represents the significant threshold 0.05. (a) Tissue type of SNP heritability enrichment of 25 tissues in MDD. (b) Tissue type of SNP heritability enrichment of 25 tissues in acute appendicitis. (c) Tissue type of SNP heritability enrichment of 25 tissues in irritable bowel syndrome. (d) Tissue type of SNP heritability enrichment of 25 tissues in ulcer of esophagus. MDD, major depression disorder; SNP, single‐nucleotide polymorphism.

Based on the result of MR, we further defined the partitioned heritability of MDD and three GI diseases by S‐LDSC (Figure 4 and Table S6). For MDD, there were five significant tissue types of SNP heritability enrichment when using top 10% of marker genes and the most significant tissue was liver, while six significant results when using top 5% of marker genes and the most significant tissue was brain cortex. For acute appendicitis, there were four significant tissue types of SNP heritability enrichment when using top 10% of marker genes and the most significant tissue was esophagus gastroesophageal junction, while three significant results when using top 5% of marker genes and the most significant tissue was colon sigmoid. For IBS, there was only one significant tissue type of SNP heritability enrichment when using top 10% as well as top 5% of marker genes. For ulcer of esophagus, there were no significant tissue type of SNP heritability enrichment when using top 10% as well as top 5% of marker genes. However, there were no significant overlapping tissues between MDD and GI diseases.

### 3.4. Summary for Shared Genetics Between Significant Trait Pairs

Based on the results of MR, we performed the TWAS analysis for MDD and three GI diseases. Based on the FDR, we defined 57 significant genes for MDD, 218 significant genes for acute appendicitis, one significant gene for IBS, and one significant gene for ulcer of esophagus (Figure 5 and Table S7). Furthermore, we found that MDD and acute appendicitis have three shared genes: *PRSS16, ZNF602P,* and *ZNF204P* (Table 2).

Figure 5
*Z* score of TWAS of the same genes of MDD and acute appendicitis in different tissues. (a) The expression of genes *PRSS16*, *ZNF204P*, and *ZNF602P* of MDD in 25 tissues. (b) The expression of genes *PRSS16*, *ZNF204P*, and *ZNF602P* of acute appendicitis in 25 tissues. The blue color means that the *Z* score has a negative value, while the red color means that the *Z* score has a positive value. The higher absolute value of *Z* represents stronger expression of genes. The asterisk represents that the value of *P* after the FDR of the TWAS *Z* score is significant for this gene in tissues. MDD, major depression disorder; TWAS, transcriptome‐wide association study.

**Figure:**
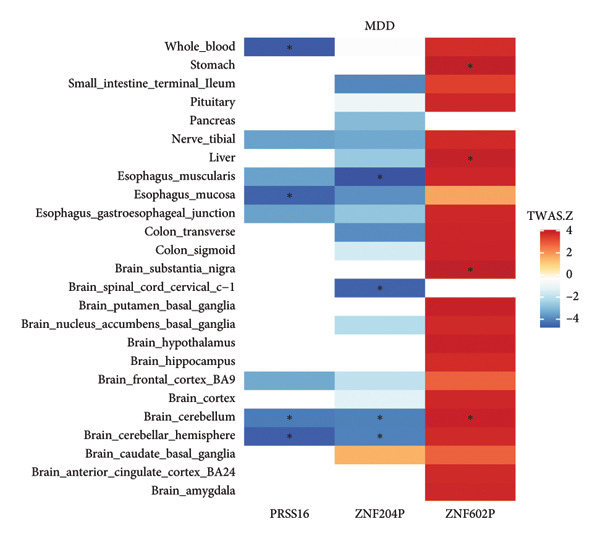
(a)

**Figure:**
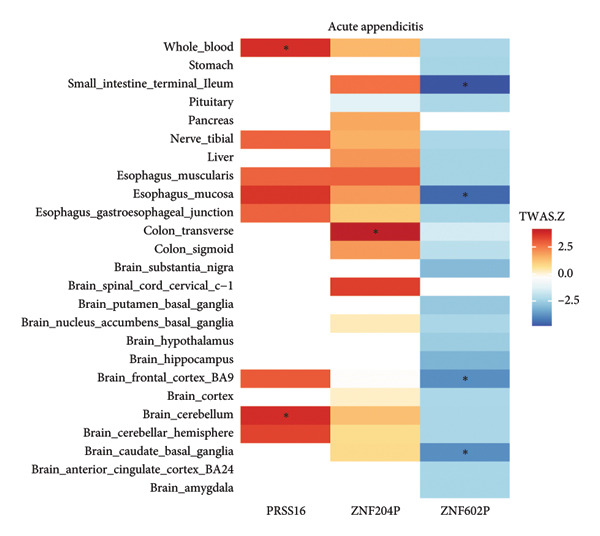
(b)

**Table 2:** The shared genes of TWAS for MDD and acute appendicitis.

Disease	Gene	Tissue	*p*	FDR
MDD	*PRSS16*	Brain cerebellar hemisphere	3.82 × 10^−6^	0.006
MDD	*PRSS16*	Brain cerebellum	3.70 × 10^−5^	0.028
MDD	*PRSS16*	Esophagus mucosa	5.28 × 10^−6^	0.026
MDD	*PRSS16*	Whole blood	3.06 × 10^−6^	0.024
Acute appendicitis	*PRSS16*	Brain cerebellum	1.81 × 10^−4^	0.042
Acute appendicitis	*PRSS16*	Whole blood	1.97 × 10^−4^	0.046
MDD	*ZNF602P*	Brain cerebellum	9.87 × 10^−5^	0.042
MDD	*ZNF602P*	Brain substantia nigra	4.71 × 10^−5^	0.031
MDD	*ZNF602P*	Liver	7.83 × 10^−5^	0.046
MDD	*ZNF602P*	Stomach	5.76 × 10^−5^	0.048
Acute appendicitis	*ZNF602P*	Brain caudate basal ganglia	1.18 × 10^−4^	0.038
Acute appendicitis	*ZNF602P*	Brain frontal cortex	1.18 × 10^−4^	0.028
Acute appendicitis	*ZNF602P*	Esophagus mucosa	6.66 × 10^−6^	0.006
Acute appendicitis	*ZNF602P*	Small intestine terminal ileum	1.52 × 10^−6^	0.001
MDD	*ZNF204P*	Brain cerebellar hemisphere	5.78 × 10^−5^	0.035
MDD	*ZNF204P*	Brain cerebellum	5.11 × 10^−5^	0.031
MDD	*ZNF204P*	Brain spinal cord cervical c‐1	5.36 × 10^−6^	0.008
MDD	*ZNF204P*	Esophagus muscularis	2.02 × 10^−6^	0.009
Acute appendicitis	*ZNF204P*	Colon transverse	2.99 × 10^−5^	0.012

After TWAS, we further performed the colocalization analysis to find whether there were shared pleiotropic genes between MDD and three GI diseases. Based on the FDR, we defined 26 significant pleiotropic genes for MDD, and two significant pleiotropic genes for acute appendicitis when using candidate genes of TWAS of MDD. We also defined 23 significant pleiotropic genes for MDD and 96 significant pleiotropic genes for acute appendicitis when using candidate genes of TWAS of acute appendicitis. Besides, we defined one pleiotropic gene for IBS when using candidate genes of TWAS of IBS and one pleiotropic gene for ulcer of esophagus when using candidate genes of TWAS of ulcer of esophagus (Table S8). And we found that five genes *C4A*, *FLOT1*, *LINC00243*, *MICB,* and *PRSS16* shared the same tissues between MDD and acute appendicitis (Table 3).

**Table 3:** The shared pleiotropic genes and SNPs of colocalization for MDD and acute appendicitis.

Disease	Tissue	PP.H4	Lead SNP	Gene
MDD	Brain amygdala	0.856	rs2534684	*MICB*
Acute appendicitis	Brain amygdala	0.881	rs1980496	*MICB*
MDD	Brain anterior cingulate cortex	0.776	rs2894221	*MICB*
Acute appendicitis	Brain anterior cingulate cortex	0.982	rs3130924	*MICB*
MDD	Brain caudate basal ganglia	0.940	rs2534684	*MICB*
Acute appendicitis	Brain caudate basal ganglia	0.933	rs3130924	*MICB*
MDD	Brain hypothalamus	0.931	rs2534684	*MICB*
Acute appendicitis	Brain hypothalamus	0.812	rs2894221	*MICB*
MDD	Brain putamen basal ganglia	0.936	rs2534684	*MICB*
Acute appendicitis	Brain putamen basal ganglia	0.861	rs3130924	*MICB*
MDD	Colon transverse	0.844	rs3130660	*FLOT1*
Acute appendicitis	Colon transverse	0.783	rs3095151	*FLOT1*
MDD	Esophagus mucosa	0.951	rs3130660	*LINC00243*
Acute appendicitis	Esophagus mucosa	0.891	rs3131934	*LINC00243*
MDD	Esophagus mucosa	0.962	rs34388707	*PRSS16*
Acute appendicitis	Esophagus mucosa	0.817	rs34569203	*PRSS16*
MDD	Pituitary	0.927	rs2534684	*MICB*
Acute appendicitis	Pituitary	0.935	rs3130924	*MICB*
MDD	Stomach	0.905	rs3094125	*FLOT1*
Acute appendicitis	Stomach	0.873	rs3131934	*FLOT1*

## 4. Discussion

In our study, we conducted an integrated analysis to explore the shared genetic basis between MDD and eight GI diseases. For the six GI diseases genetically correlating to MDD, we further performed two‐sample MR analysis to examine their causal relationship. Based on multiple testing correction and a series of sensitivity analyses, as well as reverse‐direction MR, we defined a positive causal relationship between MDD and acute appendicitis (OR = 1.09), IBS (OR = 1.14), and ulcer of esophagus (OR = 1.24). Subsequently, we conducted S‐LDSC and TWAS to explore the expression of genes of MDD and the three GI diseases across various tissues. TWAS revealed three overlapping genes between MDD and acute appendicitis. Finally, the colocalization analysis identified five shared pleiotropic genes for MDD and acute appendicitis: *C4A*, *FLOT1*, *LINC00243*, *MICB,* and *PRSS16*.

Our results suggest a potential causal association between MDD and an increased risk of IBS and ulcer of esophagus, which is consistent with findings from previous studies [18, 42, 43]. Moreover, we uncovered a causal relationship between MDD and acute appendicitis—a novel finding that requires further validation. In this analysis, given the relatively weak signals observed in the GWAS summary statistics and the limited number of available IVs, we employed a more lenient *p* value threshold of 1 × 10^−5^, rather than the conventional level of 5 × 10^−8^. While this relaxed threshold may increase the likelihood of false‐positive findings, we performed three sensitivity analyses as well as multiple testing corrections to enhance the robustness of our results and to balance statistical power with control of Type I error.

The direction of the effect we observed (i.e., MDD increasing appendicitis risk) appears to contradict previous research that suggested a potential protective effect of manic‐depressive psychosis against acute appendicitis [44]. This discrepancy could be attributed to several factors, including fundamental differences in phenotype definition (MDD vs. depression), the genetic nature of our analysis versus the observational design of prior work, or the utilization of distinct datasets and populations. Therefore, our results should not be interpreted as definitive proof of causation but rather as robust preliminary genetic evidence highlighting a previously unexplored relationship. Consequently, future investigations employing larger, more powerful datasets, such as the UK Biobank, and more stringent genetic instruments are essential to confirm or refute this putative causal link.

In our TWAS analysis, we used eQTLs from the GTEx database as reference panels. Unlike other TWAS studies that focus on identifying candidate genes, we used TWAS to investigate gene expression associated with specific traits in different tissues. We identified three shared genes—*PRSS16*, *ZNF602P*, and *ZNF204P*—between MDD and acute appendicitis. The gene *ZNF204P*, also known as zinc finger protein 204, functions as a pseudogene. A study has revealed its involvement as a long noncoding RNA in hepatocellular carcinoma development [45]. Similarly, *ZNF602P*, or zinc finger protein 602, also functions as a pseudogene. *PRSS16*, or serine protease 16, plays an important role in human life processes and has been identified as a risk gene for schizophrenia [46]. However, the precise association between *PRSS16* and MDD, as well as acute appendicitis, remains to be confirmed.

Additionally, through the colocalization analysis, we identified five shared pleiotropic genes between MDD and acute appendicitis: *C4A*, *FLOT1*, *LINC00243*, *MICB*, and *PRSS16*. *C4A*, also called Complement *C4A*, plays a key role in the human immune system and has established links with depression [47] and IBD [48]. *FLOT1*, or Flotillin 1, has been confirmed as a significant contributor to MDD and GI malignancy [49], although its specific role in other GI diseases remains to be studied. *LINC00243*, also known as long intergenic nonprotein coding RNA 243, is involved in the development of certain cancers [50]. *MICB*, or MHC Class I Polypeptide‐Related Sequence B, is crucial for GI tract function [51] and has recently been identified as a risky gene for depression among perinatal populations [52]. In total, the genes identified in our study provide new insights into the shared genetic mechanisms underlying MDD and acute appendicitis.

Indeed, previous studies have demonstrated that MDD can impact gut microbiota, which in turn affects GI health [53, 54]. Additionally, the microbiota–gut–brain axis has been shown to mediate the relationship between MDD and GI diseases [55]. However, most of these studies have primarily relied on indirect evidence. Unlike these studies, our research provides direct evidence of this connection. We have not only confirmed the causal relationship between MDD and three specific GI diseases, but have also identified shared genes between MDD and acute appendicitis. Our findings provide the basis for public health policies, underscoring the need for medical professionals to closely monitor the GI health of individuals diagnosed with MDD, with particular attention to potential emergencies.

Compared with previous studies, our study not only expands the types of GI diseases studied but also uses a new combination of methods. The major strength of our study lies in its multifaceted analytical approach, providing a robust framework for understanding the complex genetic overlaps that link MDD and GI diseases. Specifically, LDSC determines the genetic correlation between traits, while MR analyses identify the causal relationship between MDD and GI diseases. Furthermore, S‐LDSC explores the possible gene expression of the trait. TWAS facilitates the exploration of gene expression in various tissues, and the colocalization analysis helps identify shared causal genetic variants. The integration of these methods enables us not only to identify causality but also to search for pleiotropic genes, thus offering valuable insights into potential therapeutic targets and directions for future research.

Our findings, within the framework of MR and its core assumptions, provide evidence supporting a potential causal role of MDD on the risk of acute appendicitis, IBS, and esophageal ulcer. It is important to emphasize that the causal estimates from MR are valid only under the satisfaction of the three key instrumental variable assumptions that the genetic variants are strongly associated with the exposure (MDD), are not associated with any confounders of the exposure‐outcome relationship, and influence the outcome solely through the exposure (no horizontal pleiotropy). While we employed multiple sensitivity analyses to mitigate violations of these assumptions, particularly pleiotropy, the interpretability of our results remains contingent upon these assumptions holding true.

There are certain limitations to our study. First, we did not aggregate all datasets covering relevant traits, such as the UK Biobank, which may affect the robustness of our findings due to smaller sample sizes. Furthermore, while we employed TWAS to identify gene expression, we did not subsequently validate these genes. Additionally, our colocalization analysis was restricted to genes identified through TWAS, potentially overlooking other significant genetic variants. In addition, we chose FDR correction instead of Bonferroni correction for multiple testing, which could increase the risk of false positives. The use of a lenient *p* value threshold (1*e* − 5) for IV selection, while necessary for power, might have introduced weak instrument bias or increased false positives, and the estimates should be interpreted with this caveat in mind. Although we mitigated this through sensitivity analyses, the possibility of residual bias cannot be fully excluded. Lastly, our study only analyzed the individuals of European descent. Therefore, the generalizability of our results to populations of different ancestries is limited. Furthermore, our findings represent the lifelong risk associated with genetic predisposition to MDD, as genetic instruments are fixed at birth. This may not directly generalize to the risk posed by MDD episodes occurring at specific life stages. Similarly, it remains to be tested whether the observed genetic correlations and causal relationships are consistent across different age groups (e.g., younger vs. older adults) or other population strata defined by sex or socioeconomic status. Future studies in diverse cohorts are necessary to assess the universality of the gut–brain axis mechanisms identified here.

## 5. Conclusion

In conclusion, our study provides compelling evidence for the genetic association between MDD and GI diseases, particularly with acute appendicitis. We have identified shared pleiotropic genes between MDD and acute appendicitis, offering new insights into their shared genetic basis. Additionally, our findings could guide further research into the etiology and prognosis of acute appendicitis, potentially paving the way for novel therapeutic strategies.

## Ethics Statement

The authors have nothing to report.

## Consent

The authors have nothing to report.

## Disclosure

All co‐authors have seen and agreed with the manuscript’s contents.

## Conflicts of Interest

The authors declare no conflicts of interest.

## Author Contributions

Conceptualization and supervision: Fu Sun, Peng Huang, and Sheng Yang. Data curation: Fu Sun, Haochang Li, Shirong Hui, Ran He, Yihao Li, and Meijie Yu. Formal analysis and visualization: Haochang Li, Bin Gong, Shirong Hui, and Ran He. Validation and writing–original draft: Fu Sun, Bin Gong, and Haochang Li. Writing–review and editing: Fu Sun, Haochang Li, Peng Huang, and Sheng Yang. Fu Sun and Haochang Li contributed equally to this study.

## Funding

No funding was received for this research.

## Supporting Information

Table S1: The results of LDSC.

Table S2: The summary information about IVs.

Table S3: The results of MR.

Table S4: The results of the sensitivity analysis for direct MR.

Table S5: The results of the sensitivity analysis for reverse MR.

Table S6: The results of S‐LDSC.

Table S7: The results of TWAS.

Table S8: The results of colocalization.

Supporting Information 1: The results of direct MR.

Supporting Information 2: The results of reverse MR.

## Supporting information


**Supporting Information** Additional supporting information can be found online in the Supporting Information section.

## Data Availability

Data sharing is not applicable to this article as no datasets were generated or analyzed during the current study.

## References

[1] Black C. J. , Drossman D. A. , Talley N. J. , Ruddy J. , and Ford A. C. , Functional Gastrointestinal Disorders: Advances in Understanding and Management, The Lancet. (2020) 396, no. 10263, 1664–1674, 10.1016/s0140-6736(20)32115-2. 33049221

[2] Mittermaier C. , Dejaco C. , Waldhoer T. et al., Impact of Depressive Mood on Relapse in Patients With Inflammatory Bowel Disease: A Prospective 18-Month Follow-Up Study, Psychosomatic Medicine. (2004) 66, no. 1, 79–84, 10.1097/01.psy.0000106907.24881.f2, 2-s2.0-0742270563. 14747641

[3] Bernstein C. N. , Singh S. , Graff L. A. , Walker J. R. , Miller N. , and Cheang M. , A Prospective Population-Based Study of Triggers of Symptomatic Flares in IBD, American Journal of Gastroenterology. (2010) 105, no. 9, 1994–2002, 10.1038/ajg.2010.140, 2-s2.0-77956343420. 20372115

[4] Koloski N. A. , Jones M. , Kalantar J. , Weltman M. , Zaguirre J. , and Talley N. J. , The Brain--Gut Pathway in Functional Gastrointestinal Disorders is Bidirectional: A 12-Year Prospective Population-Based Study, Gut. (2012) 61, no. 9, 1284–1290, 10.1136/gutjnl-2011-300474, 2-s2.0-84860649408. 22234979

[5] Sibelli A. , Chalder T. , Everitt H. , Workman P. , Windgassen S. , and Moss-Morris R. , A Systematic Review With Meta-Analysis of the Role of Anxiety and Depression in Irritable Bowel Syndrome Onset, Psychological Medicine. (2016) 46, no. 15, 3065–3080, 10.1017/s0033291716001987, 2-s2.0-84986570889. 27605134

[6] He M. , Wang Q. , Yao D. , Li J. , and Bai G. , Association Between Psychosocial Disorders and Gastroesophageal Reflux Disease: A Systematic Review and Meta-Analysis, Journal of Neurogastroenterology and Motility. (2022) 28, no. 2, 212–221, 10.5056/jnm21044. 35362447 PMC8978133

[7] Kim S. Y. , Min C. , Oh D. J. , and Choi H. G. , Reciprocal Association Between Depression and Peptic Ulcers: Two Longitudinal Follow-Up Studies Using a National Sample Cohort, Scientific Reports. (2020) 10, no. 1, 10.1038/s41598-020-58783-0. PMC7000829 32020020

[8] Boyko E. J. , Observational Research--Opportunities and Limitations, Journal of Diabetes and Its Complications. (2013) 27, no. 6, 642–648, 10.1016/j.jdiacomp.2013.07.007, 2-s2.0-84887036209. 24055326 PMC3818421

[9] Abdellaoui A. , Yengo L. , Verweij K. J. H. , and Visscher P. M. , 15 Years of GWAS Discovery: Realizing the Promise, The American Journal of Human Genetics. (2023) 110, no. 2, 179–194, 10.1016/j.ajhg.2022.12.011. 36634672 PMC9943775

[10] Tam V. , Patel N. , Turcotte M. , Bossé Y. , Paré G. , and Meyre D. , Benefits and Limitations of Genome-Wide Association Studies, Nature Reviews Genetics. (2019) 20, no. 8, 467–484, 10.1038/s41576-019-0127-1, 2-s2.0-85065547355. 31068683

[11] Wray N. R. , Ripke S. , Mattheisen M. et al., Genome-Wide Association Analyses Identify 44 Risk Variants and Refine the Genetic Architecture of Major Depression, Nature Genetics. (2018) 50, no. 5, 668–681, 10.1038/s41588-018-0090-3, 2-s2.0-85046022254. 29700475 PMC5934326

[12] Howard D. M. , Adams M. J. , Clarke T. K. et al., Genome-Wide Meta-Analysis of Depression Identifies 102 Independent Variants and Highlights the Importance of the Prefrontal Brain Regions, Nature Neuroscience. (2019) 22, no. 3, 343–352, 10.1038/s41593-018-0326-7, 2-s2.0-85061198174. 30718901 PMC6522363

[13] Bulik-Sullivan B. K. , Loh P. R. , Finucane H. K. et al., LD Score Regression Distinguishes Confounding From Polygenicity in Genome-Wide Association Studies, Nature Genetics. (2015) 47, no. 3, 291–295, 10.1038/ng.3211, 2-s2.0-84923946495. 25642630 PMC4495769

[14] Finucane H. K. , Bulik-Sullivan B. , Gusev A. et al., Partitioning Heritability by Functional Annotation Using Genome-Wide Association Summary Statistics, Nature Genetics. (2015) 47, no. 11, 1228–1235, 10.1038/ng.3404, 2-s2.0-85000443086. 26414678 PMC4626285

[15] Skrivankova V. W. , Richmond R. C. , Woolf B. A. R. et al., Strengthening the Reporting of Observational Studies in Epidemiology Using Mendelian Randomisation (STROBE-MR): Explanation and Elaboration, BMJ. (2021) 375, 10.1136/bmj.n2233. PMC8546498 34702754

[16] Gusev A. , Ko A. , Shi H. et al., Integrative Approaches for Large-Scale Transcriptome-Wide Association Studies, Nature Genetics. (2016) 48, no. 3, 245–252, 10.1038/ng.3506, 2-s2.0-84959547986. 26854917 PMC4767558

[17] Wu Y. , Broadaway K. A. , Raulerson C. K. et al., Colocalization of GWAS and eQTL Signals at Loci With Multiple Signals Identifies Additional Candidate Genes for Body Fat Distribution, Human Molecular Genetics. (2019) 28, no. 24, 4161–4172, 10.1093/hmg/ddz263. 31691812 PMC7202621

[18] Ruan X. , Chen J. , Sun Y. et al., Depression and 24 Gastrointestinal Diseases: A Mendelian Randomization Study, Translational Psychiatry. (2023) 13, no. 1, 10.1038/s41398-023-02459-6. PMC10160129 37142593

[19] Gong W. , Guo P. , Li Y. et al., Role of the Gut-Brain Axis in the Shared Genetic Etiology Between Gastrointestinal Tract Diseases and Psychiatric Disorders: A Genome-Wide Pleiotropic Analysis, JAMA Psychiatry. (2023) 80, no. 4, 360–370, 10.1001/jamapsychiatry.2022.4974. 36753304 PMC9909581

[20] Kurki M. I. , Karjalainen J. , Palta P. et al., FinnGen Provides Genetic Insights From a Well-Phenotyped Isolated Population, Nature. (2023) 613, no. 7944, 508–518, 10.1038/s41586-022-05473-8. 36653562 PMC9849126

[21] Yang S. and Zhou X. , PGS-server: Accuracy, Robustness and Transferability of Polygenic Score Methods for Biobank Scale Studies, Briefings in Bioinformatics. (2022) 23, no. 2, 10.1093/bib/bbac039. 35193147

[22] Cao C. , Zhang S. , Wang J. et al., PGS-Depot: A Comprehensive Resource for Polygenic Scores constructed by Summary Statistics Based Methods, Nucleic Acids Research. (2024) 52, no. D1, D963–d971, 10.1093/nar/gkad1029. 37953384 PMC10767792

[23] Cao C. , Tian M. , Li Z. , Zhu W. , Huang P. , and Yang S. , GWAShug: A Comprehensive Platform for Decoding the Shared Genetic Basis Between Complex Traits Based on Summary Statistics, Nucleic Acids Research. (2025) 53, no. D1, D1006–d1015, 10.1093/nar/gkae873. 39380491 PMC11701566

[24] Auton A. , Abecasis G. R. , Altshuler D. M. et al., A Global Reference for Human Genetic Variation, Nature. (2015) 526, no. 7571, 68–74, 10.1038/nature15393, 2-s2.0-84943171338. 26432245 PMC4750478

[25] Aguet F. , Anand S. , Ardlie K. G. et al., The GTEx Consortium Atlas of Genetic Regulatory Effects Across Human Tissues, Science.(2020) 369, no. 6509, 1318–1330, 10.1126/science.aaz1776. 32913098 PMC7737656

[26] Yang Y. , Musco H. , Simpson-Yap S. et al., Investigating the Shared Genetic Architecture Between Multiple Sclerosis and Inflammatory Bowel Diseases, Nature Communications. (2021) 12, no. 1, 10.1038/s41467-021-25768-0. PMC8463615 34561436

[27] Chen X. , Yao T. , Cai J. , Fu X. , Li H. , and Wu J. , Systemic inflammatory Regulators and 7 Major Psychiatric Disorders: A Two-Sample Mendelian Randomization Study, Progress in Neuro-Psychopharmacology and Biological Psychiatry. (2022) 116, 10.1016/j.pnpbp.2022.110534. 35150783

[28] Ellervik C. , Mora S. , Ridker P. M. , and Chasman D. I. , Hypothyroidism and Kidney Function: A Mendelian Randomization Study, Thyroid. (2020) 30, no. 3, 365–379, 10.1089/thy.2019.0167. 31910748 PMC7074918

[29] Huang P. , Zou Y. , Zhang X. et al., The Causal Effects of Insomnia on Bipolar Disorder, Depression, and Schizophrenia: A Two-Sample Mendelian Randomization Study, Frontiers in Genetics. (2021) 12, 10.3389/fgene.2021.763259. PMC8542855 34707645

[30] Burgess S. and Thompson S. G. , Avoiding Bias From Weak Instruments in Mendelian Randomization Studies, International Journal of Epidemiology. (2011) 40, no. 3, 755–764, 10.1093/ije/dyr036, 2-s2.0-79961177302. 21414999

[31] Bowden J. , Davey Smith G. , and Burgess S. , Mendelian randomization With Invalid Instruments: Effect Estimation and Bias Detection Through Egger Regression, International Journal of Epidemiology. (2015) 44, no. 2, 512–525, 10.1093/ije/dyv080, 2-s2.0-84936755918. 26050253 PMC4469799

[32] Bowden J. , Davey Smith G. , Haycock P. C. , and Burgess S. , Consistent Estimation in Mendelian Randomization With Some Invalid Instruments Using a Weighted Median Estimator, Genetic Epidemiology. (2016) 40, no. 4, 304–314, 10.1002/gepi.21965, 2-s2.0-84963670568. 27061298 PMC4849733

[33] Burgess S. , Scott R. A. , Timpson N. J. , Davey Smith G. , and Thompson S. G. , Using Published Data in Mendelian Randomization: A Blueprint for Efficient Identification of Causal Risk Factors, European Journal of Epidemiology. (2015) 30, no. 7, 543–552, 10.1007/s10654-015-0011-z, 2-s2.0-84943354221. 25773750 PMC4516908

[34] Hartwig F. P. , Davey Smith G. , and Bowden J. , Robust Inference in Summary Data Mendelian randomization Via the Zero Modal Pleiotropy Assumption, International Journal of Epidemiology. (2017) 46, no. 6, 1985–1998, 10.1093/ije/dyx102, 2-s2.0-85036649300. 29040600 PMC5837715

[35] Burgess S. , Butterworth A. , and Thompson S. G. , Mendelian Randomization Analysis With Multiple Genetic Variants Using Summarized Data, Genetic Epidemiology. (2013) 37, no. 7, 658–665, 10.1002/gepi.21758, 2-s2.0-84885840876. 24114802 PMC4377079

[36] Verbanck M. , Chen C. Y. , Neale B. , and Do R. , Detection of Widespread Horizontal Pleiotropy in Causal Relationships Inferred From Mendelian Randomization Between Complex Traits and Diseases, Nature Genetics. (2018) 50, no. 5, 693–698, 10.1038/s41588-018-0099-7, 2-s2.0-85045854182. 29686387 PMC6083837

[37] Wu F. , Huang Y. , Hu J. , and Shao Z. , Mendelian Randomization Study of Inflammatory Bowel Disease and Bone Mineral Density, BMC Medicine. (2020) 18, no. 1, 10.1186/s12916-020-01778-5. PMC7654011 33167994

[38] Liu X. , Miao Y. , Liu C. , Lu W. , Feng Q. , and Zhang Q. , Identification of Multiple Novel Susceptibility Genes Associated With Autoimmune Thyroid Disease, Frontiers in Immunology. (2023) 14, 10.3389/fimmu.2023.1161311. PMC10183592 37197658

[39] Wang Y. , Guo P. , Zhang Y. et al., Joint Analysis of Genetic Correlation, Mendelian Randomization and Colocalization Highlights the Bi-Directional Causal Association Between Hypothyroidism and Primary Biliary Cirrhosis, Frontiers in Genetics. (2021) 12, 10.3389/fgene.2021.753352. PMC8521021 34671386

[40] Giambartolomei C. , Vukcevic D. , Schadt E. E. et al., Bayesian Test for Colocalisation Between Pairs of Genetic Association Studies Using Summary Statistics, PLoS Genetics. (2014) 10, no. 5, 10.1371/journal.pgen.1004383, 2-s2.0-84901631426. PMC4022491 24830394

[41] Glickman M. E. , Rao S. R. , and Schultz M. R. , False Discovery Rate Control is a Recommended Alternative to Bonferroni-Type Adjustments in Health Studies, Journal of Clinical Epidemiology. (2014) 67, no. 8, 850–857, 10.1016/j.jclinepi.2014.03.012, 2-s2.0-84902848421. 24831050

[42] Mulugeta A. , Zhou A. , King C. , and Hyppönen E. , Association Between Major Depressive Disorder and Multiple Disease Outcomes: A Phenome-Wide Mendelian Randomisation Study in the UK Biobank, Molecular Psychiatry. (2020) 25, no. 7, 1469–1476, 10.1038/s41380-019-0486-1, 2-s2.0-85071076662. 31427754

[43] Chen D. , Zhang Y. , Huang T. , and Jia J. , Depression and Risk of Gastrointestinal Disorders: A Comprehensive Two-Sample Mendelian Randomization Study of European Ancestry, Psychological Medicine. (2023) 53, no. 15, 7309–7321, 10.1017/s0033291723000867. 37183395

[44] Ewald H. , Mortensen P. B. , and Mors O. , Decreased Risk of Acute Appendicitis in Patients With Schizophrenia or Manic-Depressive Psychosis, Schizophrenia Research. (2001) 49, no. 3, 287–293, 10.1016/s0920-9964(00)00161-4, 2-s2.0-0035971520. 11356589

[45] Hwang J. H. , Lee J. , Choi W. Y. et al., ZNF204P is a Stemness-Associated Oncogenic Long Non-Coding RNA in Hepatocellular Carcinoma, BMB Reports. (2022) 55, no. 6, 281–286, 10.5483/BMBRep.2022.55.6.001. 35168700 PMC9252894

[46] Girgenti M. J. , LoTurco J. J. , and Maher B. J. , ZNF804a Regulates Expression of the Schizophrenia-Associated Genes PRSS16, COMT, PDE4B, and DRD2, PLoS One. (2012) 7, no. 2, 10.1371/journal.pone.0032404, 2-s2.0-84857522446. PMC3288100 22384243

[47] Gerring Z. F. , Gamazon E. R. , and Derks E. M. , A Gene Co-Expression Network-Based Analysis of Multiple Brain Tissues Reveals Novel Genes and Molecular Pathways Underlying Major Depression, PLoS Genetics. (2019) 15, no. 7, 10.1371/journal.pgen.1008245, 2-s2.0-85070660202. PMC6658115 31306407

[48] Nissilä E. , Korpela K. , Lokki A. I. et al., C4B Gene Influences Intestinal Microbiota Through Complement Activation in Patients With Paediatric-Onset Inflammatory Bowel Disease, Clinical and Experimental Immunology. (2017) 190, no. 3, 394–405, 10.1111/cei.13040, 2-s2.0-85030178414. 28832994 PMC5680072

[49] Zhan Z. , Ye M. , and Jin X. , The Roles of FLOT1 in Human Diseases (Review), Molecular Medicine Reports. (2023) 28, no. 5, 10.3892/mmr.2023.13099. PMC10552069 37772385

[50] Feng X. and Yang S. , Long Non-Coding RNA LINC00243 Promotes Proliferation and Glycolysis in Non-Small Cell Lung Cancer Cells by Positively Regulating PDK4 Through Sponging miR-507, Molecular and Cellular Biochemistry. (2020) 463, no. 1-2, 127–136, 10.1007/s11010-019-03635-3, 2-s2.0-85074024647. 31595421

[51] Braud V. M. , Allan D. S. , and McMichael A. J. , Functions of Nonclassical MHC and Non-MHC-Encoded Class I Molecules, Current Opinion in Immunology. (1999) 11, no. 1, 100–108, 10.1016/s0952-7915(99)80018-1, 2-s2.0-0033002722. 10047540

[52] Accortt E. , Mirocha J. , Zhang D. , Kilpatrick S. J. , Libermann T. , and Karumanchi S. A. , Perinatal Mood and Anxiety Disorders: Biomarker Discovery Using Plasma Proteomics, American Journal of Obstetrics and Gynecology. (2023) 229, no. 2, 166.e1–166.e16, 10.1016/j.ajog.2023.01.012. 36649818

[53] Zhuang Z. , Yang R. , Wang W. , Qi L. , and Huang T. , Associations Between Gut Microbiota and Alzheimer’s Disease, Major Depressive Disorder, and Schizophrenia, Journal of Neuroinflammation. (2020) 17, no. 1, 10.1186/s12974-020-01961-8. PMC7532639 33008395

[54] McGuinness A. J. , Davis J. A. , Dawson S. L. et al., A systematic Review of Gut Microbiota Composition in Observational Studies of Major Depressive Disorder, Bipolar Disorder and Schizophrenia, Molecular Psychiatry. (2022) 27, no. 4, 1920–1935, 10.1038/s41380-022-01456-3. 35194166 PMC9126816

[55] Góralczyk-Bińkowska A. , Szmajda-Krygier D. , and Kozłowska E. , The Microbiota-Gut-Brain Axis in Psychiatric Disorders, International Journal of Molecular Sciences. (2022) 23, no. 19, 10.3390/ijms231911245. PMC9570195 36232548

