# Supplementary material for: Investigating the Causal Relationship and Shared Genetic Basis Between Major Depression Disorder and Eight Types of Gastrointestinal Diseases
Source: Genet Res (Camb). 2025 Dec 14;2025:6931243. doi: 10.1155/genr/6931243 (PMC12721742; doi:10.1155/genr/6931243)
Supplement: Supplementary file 1 — Supporting Information Additional supporting information can be found online in the Supporting Information section. [file GENR-2025-6931243-s001.zip › Supplementary materials 1.zip!/Supplementary materials 1/MDD on Crohn disease.pdf]

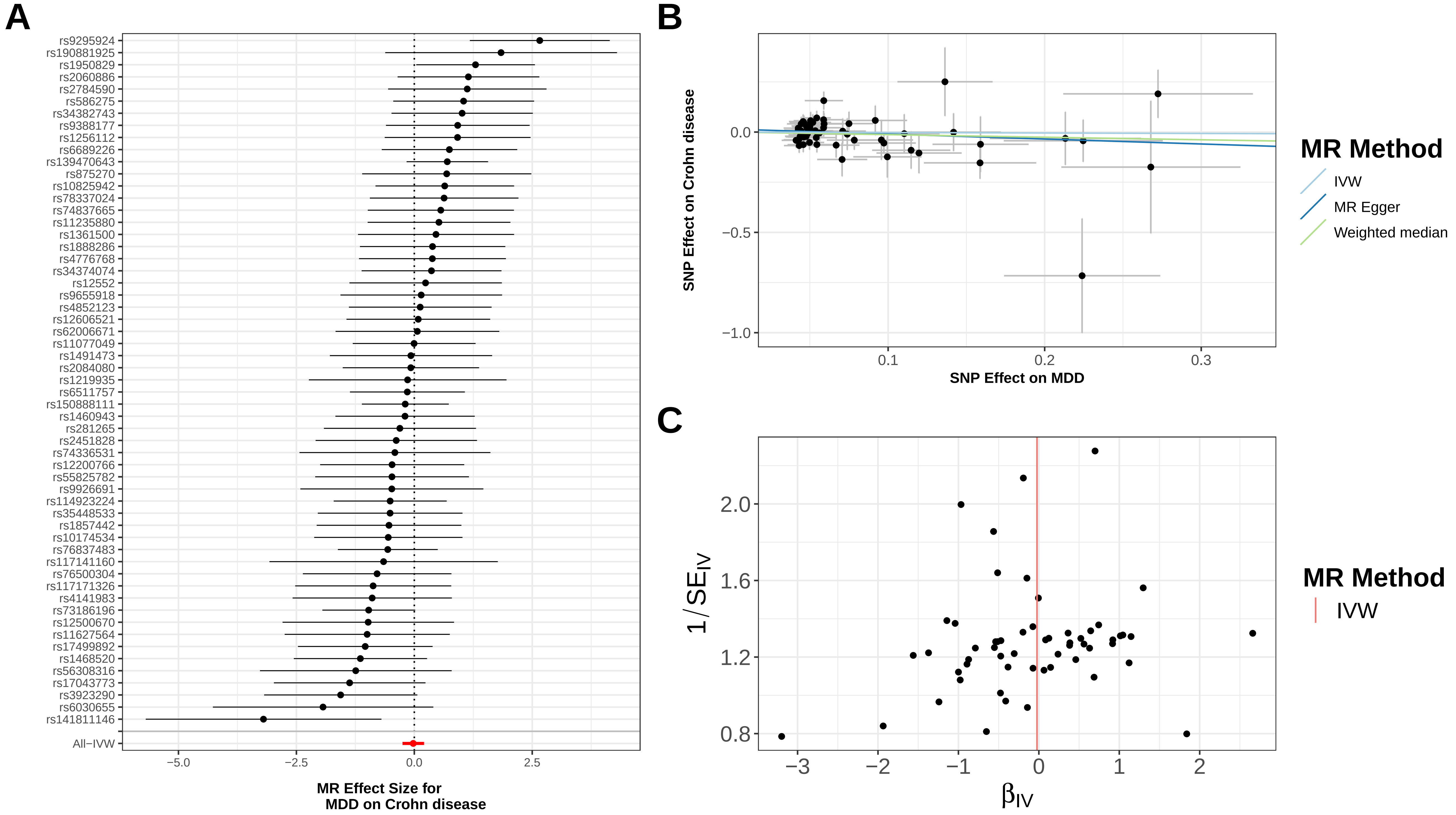
A
rs9295924
rs190881925
rs1950829
rs2060886
rs2784590
rs586275
rs34382743
rs9388177
rs1256112
rs6689226
rs139470643
rs875270
rs10825942
rs78337024
rs74837665
rs11235880
rs1361500
rs1888286
rs4776768
rs34374074
rs12552
rs9655918
rs4852123
rs12606521
rs62006671
rs11077049
rs1491473
rs2084080
rs1219935
rs6511757
rs150888111
rs1460943
rs281265
rs2451828
rs74336531
rs12200766
rs55825782
rs9926691
rs114923224
rs35448533
rs1857442
rs10174534
rs76837483
rs117141160
rs76500304
rs117171326
rs4141983
rs73186196
rs12500670
rs11627564
rs17499892
rs1468520
rs56308316
rs17043773
rs3923290
rs6030655
rs141811146
All−IVW
−5.0
−2.5
0.0
2.5
MR Effect Size for
MDD on Crohn disease
B
SNP Effect on Crohn disease
0.0
−0.5
−1.0
0.1
0.2
0.3
SNP Effect on MDD
MR Method
IVW
MR Egger
Weighted median
C
1/SE_IV
2.0
1.6
1.2
0.8
−3
−2
−1
0
1
2
β_IV
MR Method
IVW
