# Supplementary material for: Investigating the Causal Relationship and Shared Genetic Basis Between Major Depression Disorder and Eight Types of Gastrointestinal Diseases
Source: Genet Res (Camb). 2025 Dec 14;2025:6931243. doi: 10.1155/genr/6931243 (PMC12721742; doi:10.1155/genr/6931243)
Supplement: Supplementary file 1 — Supporting Information Additional supporting information can be found online in the Supporting Information section. [file GENR-2025-6931243-s001.zip › Supplementary materials 1.zip!/Supplementary materials 1/MDD on Gastric ulcer.pdf]

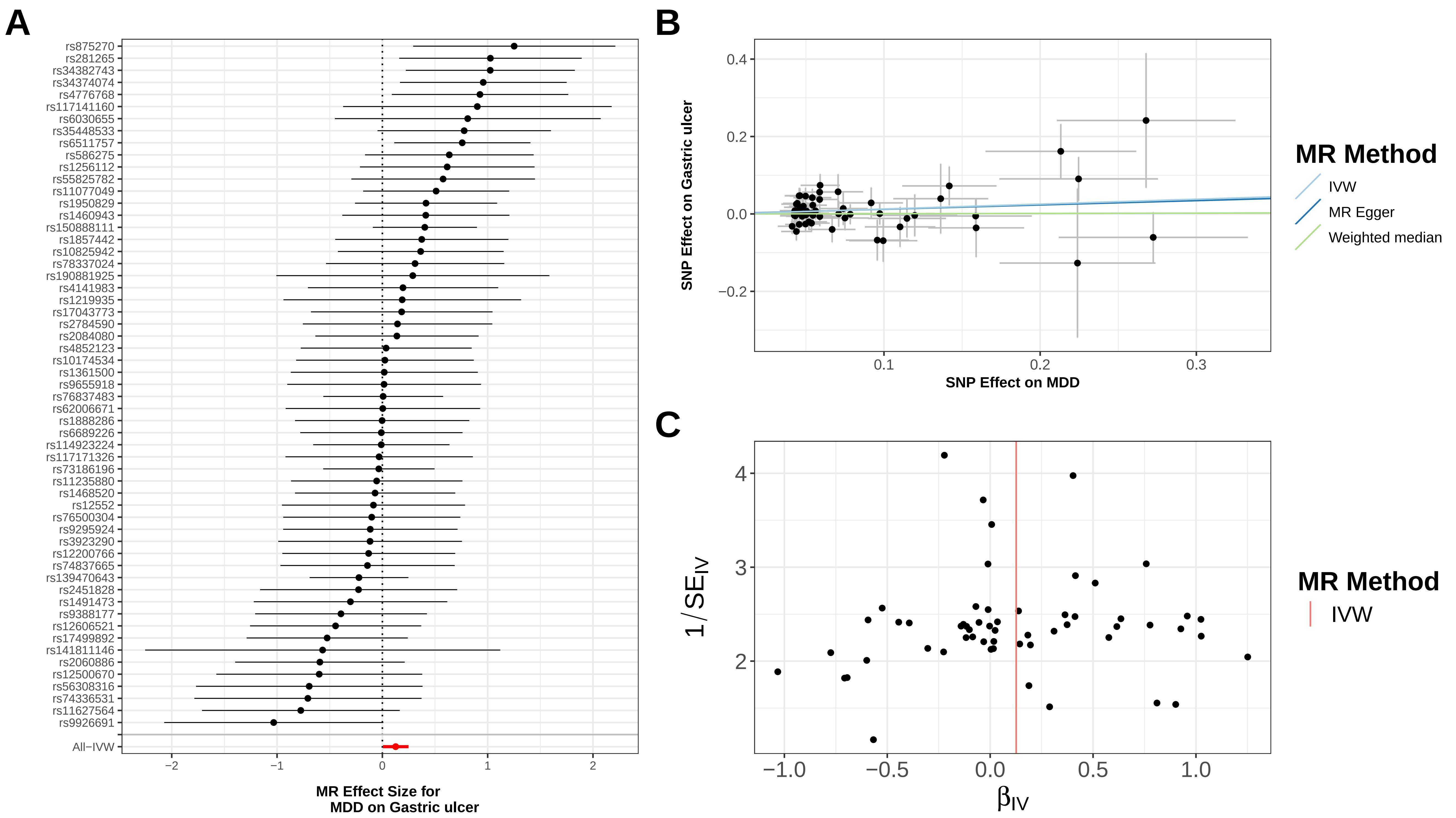
A
rs875270
rs281265
rs34382743
rs34374074
rs4776768
rs117141160
rs6030655
rs35448533
rs6511757
rs586275
rs1256112
rs55825782
rs11077049
rs1950829
rs1460943
rs150888111
rs1857442
rs10825942
rs78337024
rs190881925
rs4141983
rs1219935
rs17043773
rs2784590
rs2084080
rs4852123
rs10174534
rs1361500
rs9655918
rs76837483
rs62006671
rs1888286
rs6689226
rs114923224
rs117171326
rs73186196
rs11235880
rs1468520
rs12552
rs76500304
rs9295924
rs3923290
rs12200766
rs74837665
rs139470643
rs2451828
rs1491473
rs9388177
rs12606521
rs17499892
rs141811146
rs2060886
rs12500670
rs56308316
rs74336531
rs11627564
rs9926691
All–IVW
−2
−1
0
1
2
MR Effect Size for
MDD on Gastric ulcer
B
SNP Effect on Gastric ulcer
0.4
0.2
0.0
−0.2
0.1
0.2
0.3
SNP Effect on MDD
MR Method
IVW
MR Egger
Weighted median
C
$1/SE_{IV}$
4
3
2
−1.0
−0.5
0.0
0.5
1.0
β_{IV}
MR Method
IVW
