# Supplementary material for: Investigating the Causal Relationship and Shared Genetic Basis Between Major Depression Disorder and Eight Types of Gastrointestinal Diseases
Source: Genet Res (Camb). 2025 Dec 14;2025:6931243. doi: 10.1155/genr/6931243 (PMC12721742; doi:10.1155/genr/6931243)
Supplement: Supplementary file 1 — Supporting Information Additional supporting information can be found online in the Supporting Information section. [file GENR-2025-6931243-s001.zip › Supplementary materials 1.zip!/Supplementary materials 1/MDD on Gastroduodenal ulcer.pdf]

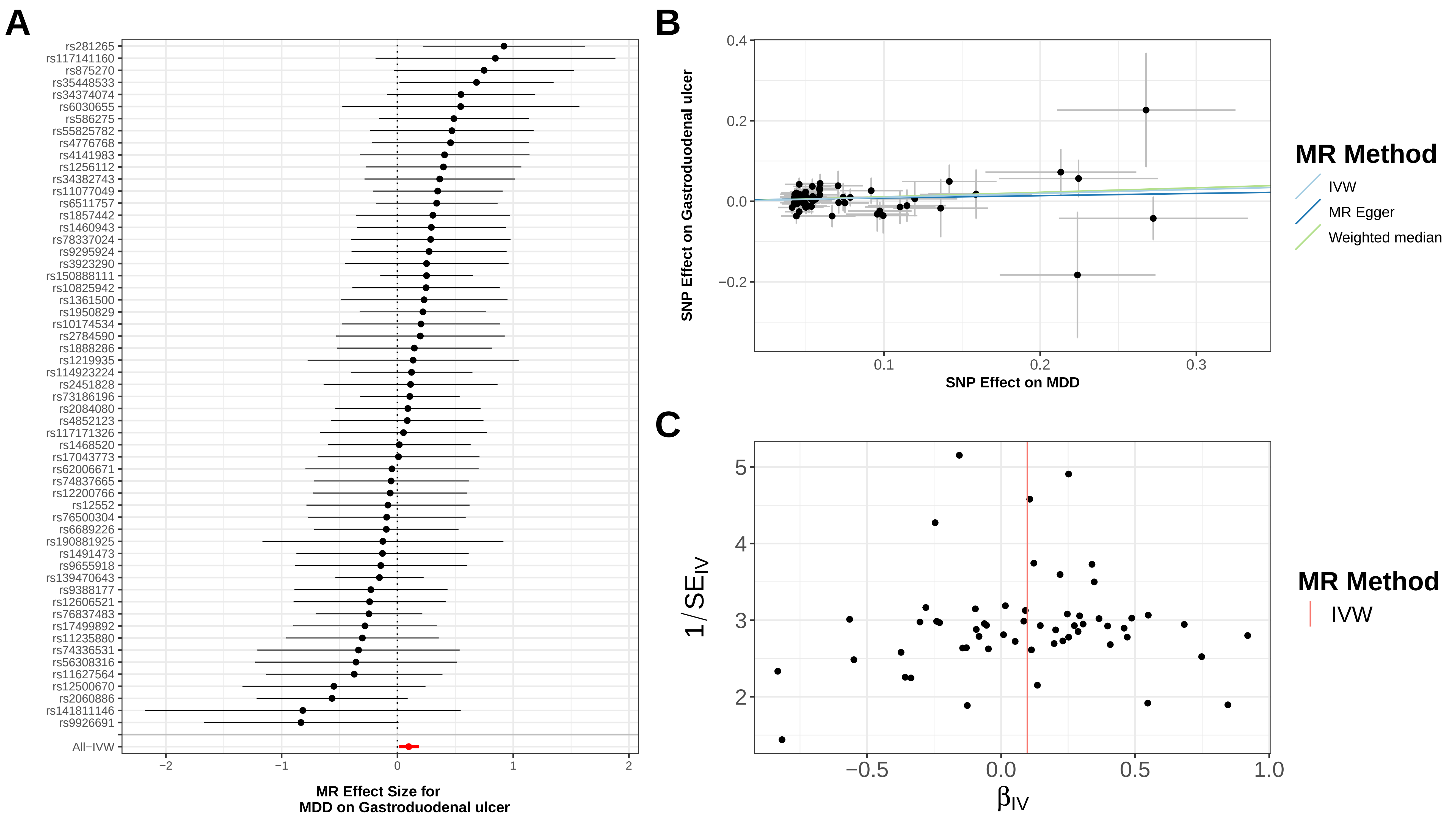

A
rs281265
rs117141160
rs875270
rs35448533
rs34374074
rs6030655
rs586275
rs55825782
rs4776768
rs4141983
rs1256112
rs34382743
rs11077049
rs6511757
rs1857442
rs1460943
rs78337024
rs9295924
rs3923290
rs150888111
rs10825942
rs1361500
rs1950829
rs10174534
rs2784590
rs1888286
rs1219935
rs114923224
rs2451828
rs73186196
rs2084080
rs4852123
rs117171326
rs1468520
rs17043773
rs62006671
rs74837665
rs12200766
rs12552
rs76500304
rs6689226
rs190881925
rs1491473
rs9655918
rs139470643
rs9388177
rs12606521
rs76837483
rs17499892
rs11235880
rs74336531
rs56308316
rs11627564
rs12500670
rs2060886
rs141811146
rs9926691
All−IVW
−2
−1
0
1
2
MR Effect Size for
MDD on Gastroduodenal ulcer
B
SNP Effect on Gastroduodenal ulcer
0.4
0.2
0.0
−0.2
0.1
0.2
0.3
SNP Effect on MDD
MR Method
IVW
MR Egger
Weighted median
C
$1/SE_{IV}$
5
4
3
2
−0.5
0.0
0.5
1.0
$\beta_{IV}$
MR Method
IVW
