# Supplementary material for: Investigating the Causal Relationship and Shared Genetic Basis Between Major Depression Disorder and Eight Types of Gastrointestinal Diseases
Source: Genet Res (Camb). 2025 Dec 14;2025:6931243. doi: 10.1155/genr/6931243 (PMC12721742; doi:10.1155/genr/6931243)
Supplement: Supplementary file 1 — Supporting Information Additional supporting information can be found online in the Supporting Information section. [file GENR-2025-6931243-s001.zip › Supplementary materials 1.zip!/Supplementary materials 1/MDD on Gastroesophageal reflux disease.pdf]

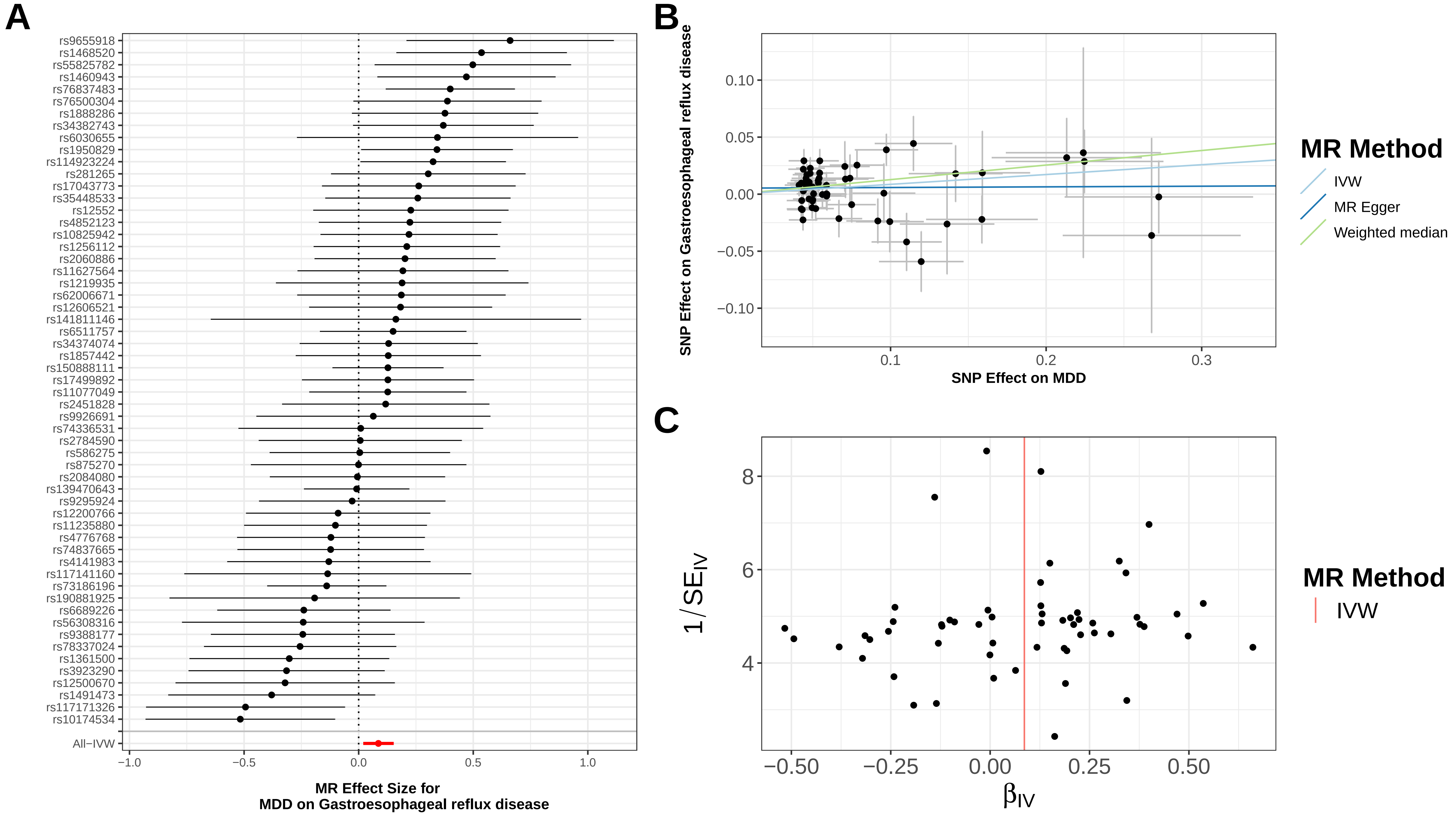
A
rs9655918
rs1468520
rs55825782
rs1460943
rs76837483
rs76500304
rs1888286
rs34382743
rs6030655
rs1950829
rs114923224
rs281265
rs17043773
rs35448533
rs12552
rs4852123
rs10825942
rs1256112
rs2060886
rs11627564
rs1219935
rs62006671
rs12606521
rs141811146
rs6511757
rs34374074
rs1857442
rs150888111
rs17499892
rs11077049
rs2451828
rs9926691
rs74336531
rs2784590
rs586275
rs875270
rs2084080
rs139470643
rs9295924
rs12200766
rs11235880
rs4776768
rs74837665
rs4141983
rs117141160
rs73186196
rs190881925
rs6689226
rs56308316
rs9388177
rs78337024
rs1361500
rs3923290
rs12500670
rs1491473
rs117171326
rs10174534
All−IVW
−1.0
−0.5
0.0
0.5
1.0
MR Effect Size for
MDD on Gastroesophageal reflux disease
B
SNP Effect on Gastroesophageal reflux disease
0.10
0.05
0.00
−0.05
−0.10
0.1
0.2
0.3
SNP Effect on MDD
MR Method
IVW
MR Egger
Weighted median
C
8
6
4
1/SE_IV
−0.50
−0.25
0.00
0.25
0.50
β_IV
MR Method
IVW
