# Supplementary material for: Investigating the Causal Relationship and Shared Genetic Basis Between Major Depression Disorder and Eight Types of Gastrointestinal Diseases
Source: Genet Res (Camb). 2025 Dec 14;2025:6931243. doi: 10.1155/genr/6931243 (PMC12721742; doi:10.1155/genr/6931243)
Supplement: Supplementary file 1 — Supporting Information Additional supporting information can be found online in the Supporting Information section. [file GENR-2025-6931243-s001.zip › Supplementary materials 1.zip!/Supplementary materials 1/MDD on Irritable bowel syndrome.pdf]

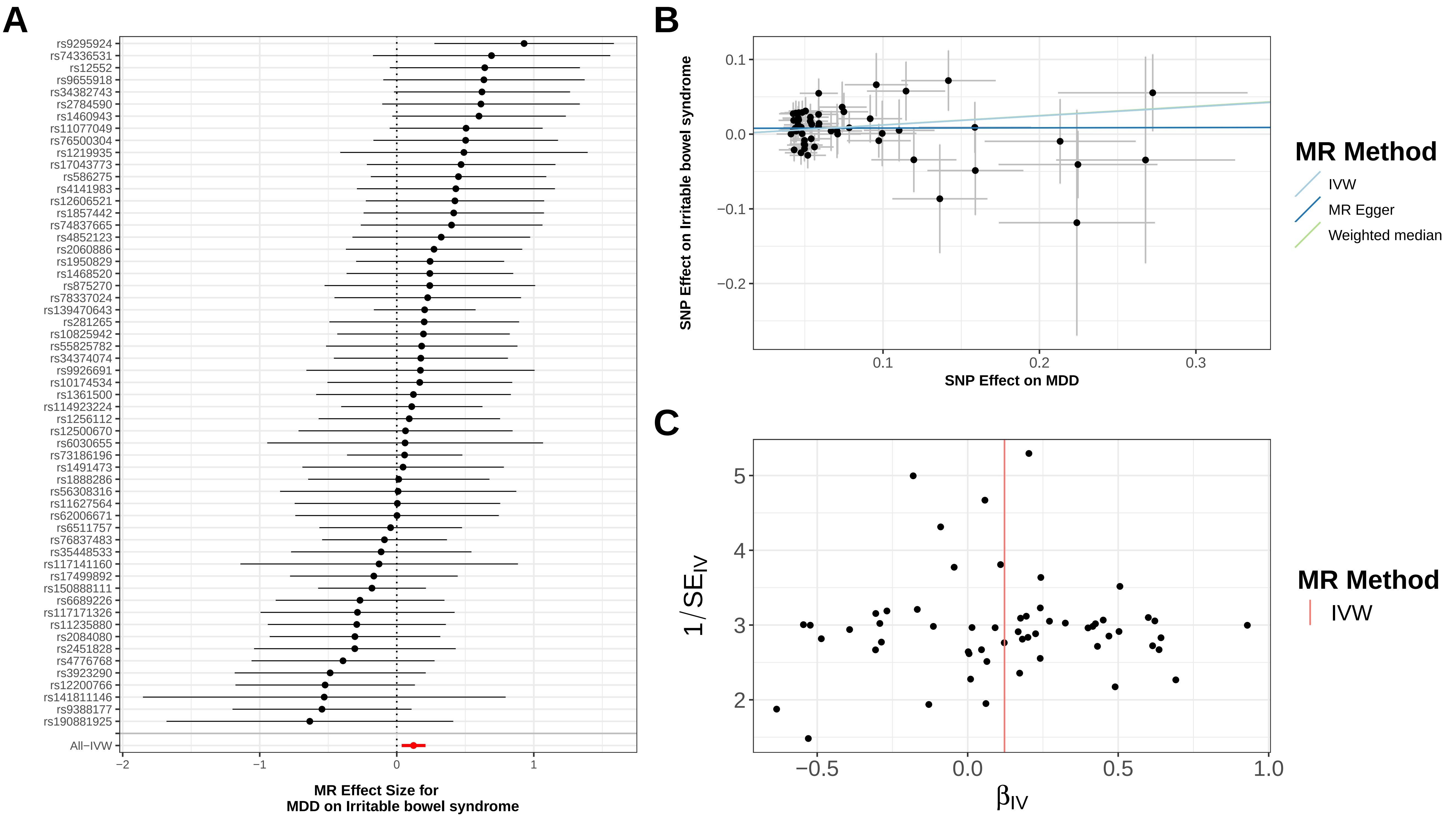
A
rs9295924
rs74336531
rs12552
rs9655918
rs34382743
rs2784590
rs1460943
rs11077049
rs76500304
rs1219935
rs17043773
rs586275
rs4141983
rs12606521
rs1857442
rs74837665
rs4852123
rs2060886
rs1950829
rs1468520
rs875270
rs78337024
rs139470643
rs281265
rs10825942
rs55825782
rs34374074
rs9926691
rs10174534
rs1361500
rs114923224
rs1256112
rs12500670
rs6030655
rs73186196
rs1491473
rs1888286
rs56308316
rs11627564
rs62006671
rs6511757
rs76837483
rs35448533
rs117141160
rs17499892
rs150888111
rs6689226
rs117171326
rs11235880
rs2084080
rs2451828
rs4776768
rs3923290
rs12200766
rs141811146
rs9388177
rs190881925
All−IVW
−2
−1
0
1
MR Effect Size for
MDD on Irritable bowel syndrome
B
SNP Effect on Irritable bowel syndrome
0.1
0.0
−0.1
−0.2
0.1
0.2
0.3
SNP Effect on MDD
MR Method
IVW
MR Egger
Weighted median
C
$1/SE_{IV}$
5
4
3
2
−0.5
0.0
0.5
1.0
β_{IV}
MR Method
IVW
