# Supplementary material for: Investigating the Causal Relationship and Shared Genetic Basis Between Major Depression Disorder and Eight Types of Gastrointestinal Diseases
Source: Genet Res (Camb). 2025 Dec 14;2025:6931243. doi: 10.1155/genr/6931243 (PMC12721742; doi:10.1155/genr/6931243)
Supplement: Supplementary file 1 — Supporting Information Additional supporting information can be found online in the Supporting Information section. [file GENR-2025-6931243-s001.zip › Supplementary materials 1.zip!/Supplementary materials 1/MDD on Ulcer of esophagus.pdf]

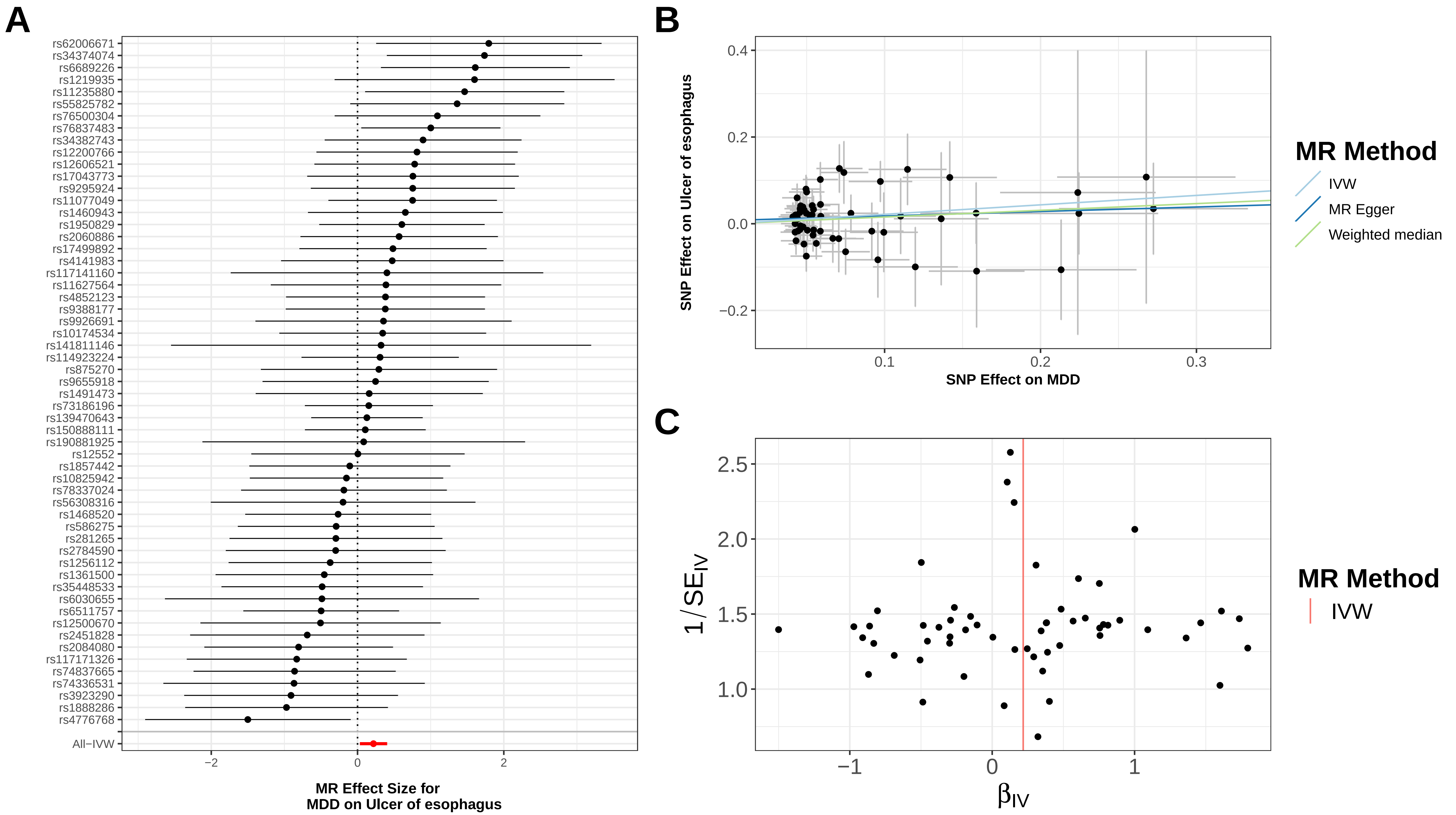

A
rs62006671
rs34374074
rs6689226
rs1219935
rs11235880
rs55825782
rs76500304
rs76837483
rs34382743
rs12200766
rs12606521
rs17043773
rs9295924
rs11077049
rs1460943
rs1950829
rs2060886
rs17499892
rs4141983
rs117141160
rs11627564
rs4852123
rs9388177
rs9926691
rs10174534
rs141811146
rs114923224
rs875270
rs9655918
rs1491473
rs73186196
rs139470643
rs150888111
rs190881925
rs12552
rs1857442
rs10825942
rs78337024
rs56308316
rs1468520
rs586275
rs281265
rs2784590
rs1256112
rs1361500
rs35448533
rs6030655
rs6511757
rs12500670
rs2451828
rs2084080
rs117171326
rs74837665
rs74336531
rs3923290
rs1888286
rs4776768
All−IVW
−2
0
2
MR Effect Size for
MDD on Ulcer of esophagus
B
0.4
0.2
0.0
−0.2
SNP Effect on Ulcer of esophagus
0.1
0.2
0.3
SNP Effect on MDD
MR Method
IVW
MR Egger
Weighted median
C
2.5
2.0
1.5
1.0
1/SE_IV
−1
0
1
β_IV
MR Method
IVW
