# Supplementary material for: Investigating the Causal Relationship and Shared Genetic Basis Between Major Depression Disorder and Eight Types of Gastrointestinal Diseases
Source: Genet Res (Camb). 2025 Dec 14;2025:6931243. doi: 10.1155/genr/6931243 (PMC12721742; doi:10.1155/genr/6931243)
Supplement: Supplementary file 1 — Supporting Information Additional supporting information can be found online in the Supporting Information section. [file GENR-2025-6931243-s001.zip › Supplementary materials 1.zip!/Supplementary materials 1/MDD on Ulcerative colitis.pdf]

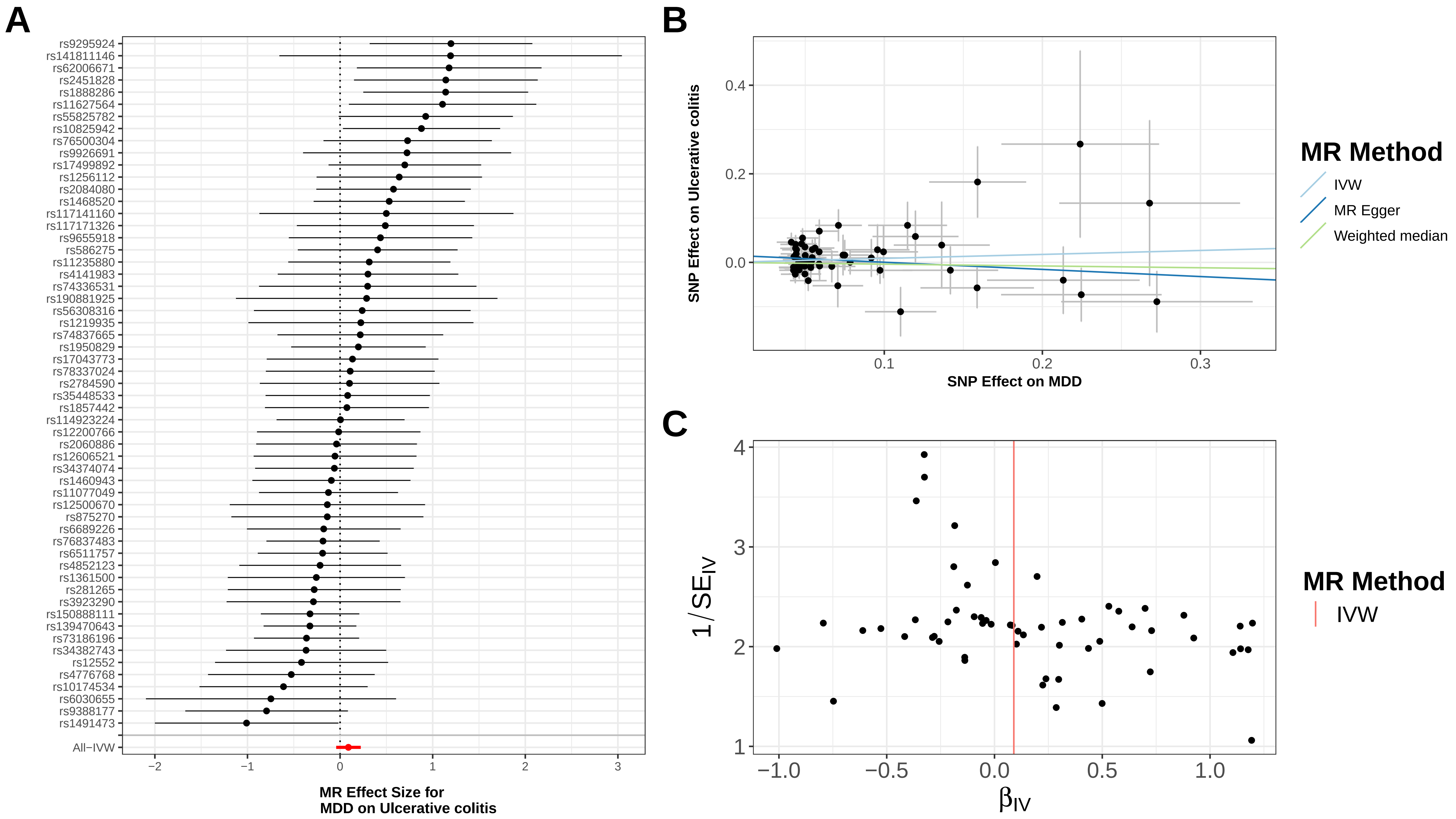
A
rs9295924
rs141811146
rs62006671
rs2451828
rs1888286
rs11627564
rs55825782
rs10825942
rs76500304
rs9926691
rs17499892
rs1256112
rs2084080
rs1468520
rs117141160
rs117171326
rs9655918
rs586275
rs11235880
rs4141983
rs74336531
rs190881925
rs56308316
rs1219935
rs74837665
rs1950829
rs17043773
rs78337024
rs2784590
rs35448533
rs1857442
rs114923224
rs12200766
rs2060886
rs12606521
rs34374074
rs1460943
rs11077049
rs12500670
rs875270
rs6689226
rs76837483
rs6511757
rs4852123
rs1361500
rs281265
rs3923290
rs150888111
rs139470643
rs73186196
rs34382743
rs12552
rs4776768
rs10174534
rs6030655
rs9388177
rs1491473
All−IVW
MR Effect Size for MDD on Ulcerative colitis
B
SNP Effect on Ulcerative colitis
SNP Effect on MDD
MR Method
IVW
MR Egger
Weighted median
C
1/SE_IV
β_IV
MR Method
IVW
