# Supplementary material for: Investigating the Causal Relationship and Shared Genetic Basis Between Major Depression Disorder and Eight Types of Gastrointestinal Diseases
Source: Genet Res (Camb). 2025 Dec 14;2025:6931243. doi: 10.1155/genr/6931243 (PMC12721742; doi:10.1155/genr/6931243)
Supplement: Supplementary file 1 — Supporting Information Additional supporting information can be found online in the Supporting Information section. [file GENR-2025-6931243-s001.zip › Supplementary materials 2.zip!/Supplementary materials 2/Crohn disease on MDD.pdf]

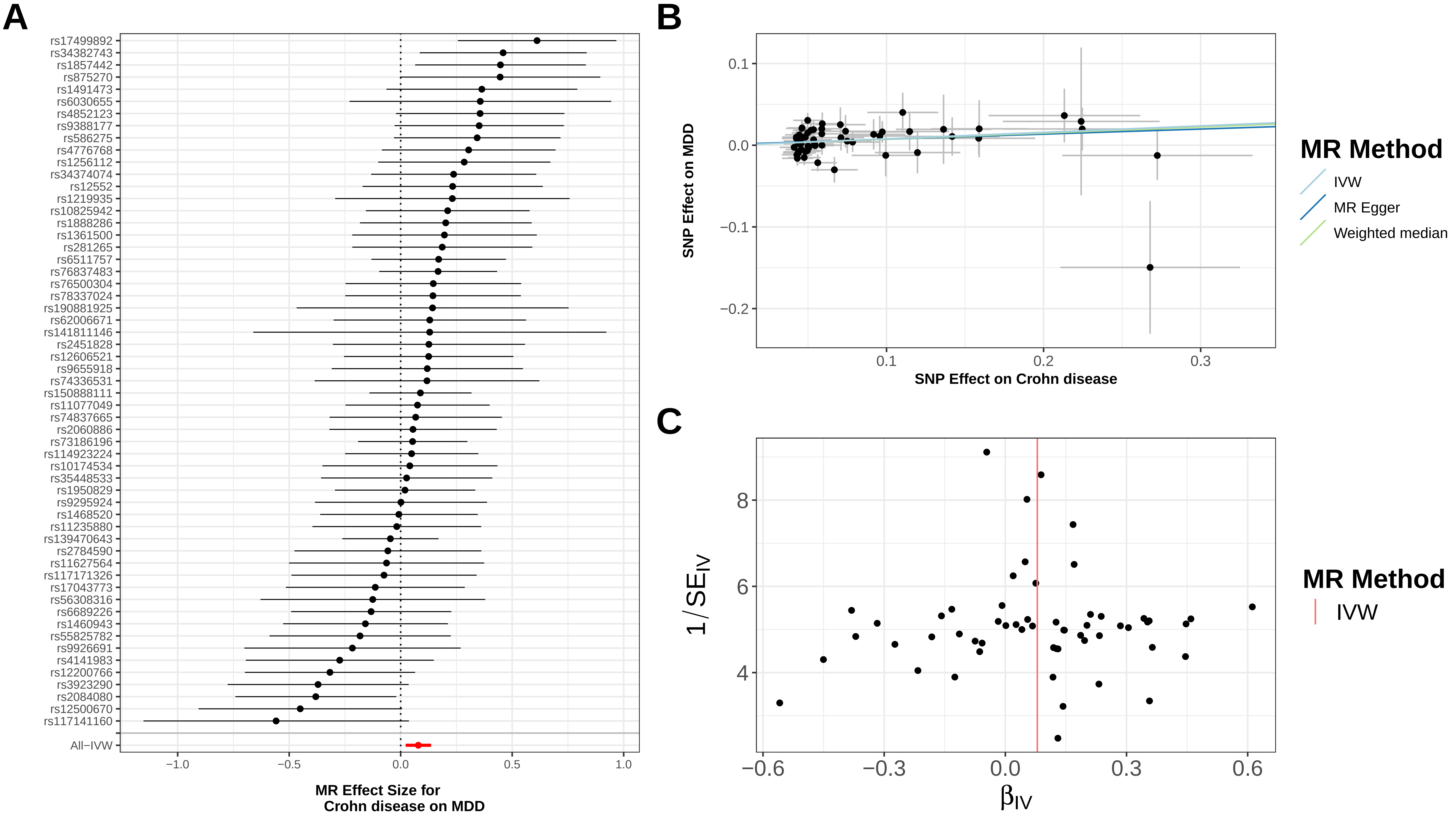
A
rs17499892
rs34382743
rs1857442
rs875270
rs1491473
rs6030655
rs4852123
rs9388177
rs586275
rs4776768
rs1256112
rs34374074
rs12552
rs1219935
rs10825942
rs1888286
rs1361500
rs281265
rs6511757
rs76837483
rs76500304
rs78337024
rs190881925
rs62006671
rs141811146
rs2451828
rs12606521
rs9655918
rs74336531
rs150888111
rs11077049
rs74837665
rs2060886
rs73186196
rs114923224
rs10174534
rs35448533
rs1950829
rs9295924
rs1468520
rs11235880
rs139470643
rs2784590
rs11627564
rs117171326
rs17043773
rs56308316
rs6689226
rs1460943
rs55825782
rs9926691
rs4141983
rs12200766
rs3923290
rs2084080
rs12500670
rs117141160
All−IVW
−1.0
−0.5
0.0
0.5
1.0
MR Effect Size for Crohn disease on MDD
B
SNP Effect on MDD
0.1
0.0
−0.1
−0.2
0.1
0.2
0.3
SNP Effect on Crohn disease
MR Method
IVW
MR Egger
Weighted median
C
$1/SE_{IV}$
8
6
4
−0.6
−0.3
0.0
0.3
0.6
β_{IV}
MR Method
IVW
